# Reconstituting neurovascular unit with primary neural stem cells and brain microvascular endothelial cells in three‐dimensional matrix

**DOI:** 10.1111/bpa.12940

**Published:** 2021-02-12

**Authors:** Hongjin Wang, Huan Yang, Yuhong Shi, Yaping Xiao, Yue Yin, Baoxiang Jiang, Huijing Ren, Weihai Chen, Qiang Xue, Xiaoyu Xu

**Affiliations:** ^1^ College of Pharmaceutical Sciences & Chinese Medicine Southwest University Chongqing China; ^2^ Chongqing Key Laboratory of New Drug Screening from Traditional Chinese Medicine Chongqing China; ^3^ Pharmacology of Chinese Materia Medica—the Key Discipline Constructed by the State Administration of Traditional Chinese Medicine Chongqing China; ^4^ Faculty of Psychology Southwest University Chongqing China; ^5^ Chongqing Medical and Pharmaceutical College Chongqing China

**Keywords:** brain microvascular endothelial cells, neural stem cells, neurovascular unit, reconstituting, three‐dimensional

## Abstract

Neurovascular dysfunction is a primary or secondary cause in the pathogenesis of several cerebrovascular and neurodegenerative disorders, including stroke. Therefore, the overall protection of the neurovascular unit (NVU) is a promising therapeutic strategy for various neurovascular diseases. However, the complexity of the NVU limits the study of the pathological mechanisms of neurovascular dysfunction. Reconstituting the in vitro NVU is important for the pathological study and drug screening of neurovascular diseases. In this study, we generated a spontaneously assembled three‐dimensional NVU (3D NVU) by employing the primary neural stem cells and brain microvascular endothelial cells in a Matrigel extracellular matrix platform. This novel model exhibits the fundamental structures and features of the NVU, including neurons, astrocytes, oligodendrocytes, vascular‐like structures, and blood–brain barrier‐like characteristics. Additionally, under oxygen‐glucose deprivation, the 3D NVU exhibits the neurovascular‐ or oxidative stress‐related pathological characteristics of cerebral ischemia and the injuries can be mitigated, respectively, by supplementing with the vascular endothelial growth factor or edaravone, which demonstrated that the availability of 3D NVU in ischemic stroke modeling. Finally, the 3D NVU promoted the angiogenesis and neurogenesis in the brain of cerebral ischemia rats. We expect that the proposed in vitro 3D NVU model will be widely used to investigate the relationships between angiogenesis and neurogenesis and to study the pathology and pharmacology of neurovascular diseases.

## Graphical abstract

The proposed 3D NVU model provides a valuable platform and precise spatiotemporal control for neurogenesis and angiogenesis research and the investigation of brain functions, which rely on interactions among brain cells, drug screening studies as a therapeutic strategy, and clinical applications (e.g., in the case of degenerative disease).

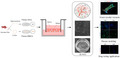

## 1 INTRODUCTION

The neurovascular unit (NVU) is an organized multicellular and multicomponent network that is important for brain health (1). This brain‐localized unit is composed of neural and vascular components, and the interface and interactions between these components are crucial for the regulation of material exchange between the bloodstream and the brain parenchyma (2, 3, 4). The neural component of the NVU consists of neurons and glial cells (microglia, astrocytes, and oligodendrocytes), while the vascular component consists of endothelial cells, pericytes, and vascular smooth muscle cells (1). The neural component—particularly the neurons—are crucial for the cognitive function of patients with various neurodegeneration diseases and are affected by a variety of pathological influences (e.g., hypoxia, oxidative stress). The vascular component—particularly the endothelial cells—forms the blood–brain barrier (BBB), which is a part of the NVU and a critical multicellular vascular structure that separates the brain from systemic blood circulation (5). Continuous intercellular junctional protein complexes between adjacent endothelium help to maintain the physical and functional integrity of the BBB, by strictly regulating the permeability through the BBB and transferring extracellular mechanical signals to the surrounding cells. The importance of vascular contributions to various brain disorders, including stroke and Alzheimer’s disease (6), is becoming increasingly recognized.

Neurovascular dysfunction is a major process in the pathogenesis of stroke and most neurodegenerative diseases. A damaged NVU leads to an impaired ability to clear toxic compounds from the brain and/or allow for the passage of harmful molecules through the BBB and into the brain (4), which may result in both the onset and progression of neurodegeneration. Despite the advances in brain imaging technology and the enhanced availability of animal models, because of the complexity of the in vivo environment, there is still an urgent need to develop cell‐based NVU in vitro models for obtaining insights into NVU and its dysfunction in related diseases. Thus, mimicking NVU function and dysfunction is essential for elucidating the physiology and pathological mechanisms underlying various neurovascular and neurodegenerative diseases. Current cell‐based BBB/NVU models are commonly classified into three categories (7). The first involves two or three cell types separated by a traditional porous physical layer, such as a transwell filter (8, 9). The second involves different types of cells in microfluidic platforms with a highly controlled, perfused environment (usually referred to as “organ‐on‐a‐chip”) (10, 11, 12, 13, 14, 15). The third involves different types of cells co‐cultured in biomaterials exhibiting the characteristics of an extracellular matrix (ECM) (usually referred to as “bioprinting”) (16, 17, 18, 19, 20).

For a long time, traditional transwell approaches have been widely adopted for modeling BBB/NVU by co‐culturing a segregated confluent monolayer of endothelial cells with different types of BBB/NVU cells in micropore membrane‐separated compartments (8, 9, 21). These are among the representative platforms for disease modeling and drug screening. Although such platforms are reproducible and easy to use, they have limitations in modeling fundamental BBB/NVU structure characteristics and microenvironmental complexities such as cell–cell and cell–matrix interactions, compromising their ability to accurately mimic brain vessels with regard to the junctional proteins and membrane transporter expression (22, 23, 24). Recently, microfluidic technology has emerged as a promising tool for reconstituting the BBB/NVU because of several advantages: microfluidic systems allow accurate control of the cellular and ECM microenvironment, while providing a platform for practical application of cellular biological responses to various stimuli (e.g., drug testing and signal transduction) (12). However, these systems fail to capture the BBB/NVU vasculature and neural‐component morphology via spontaneous programming of cell–cell interactions, as well as physiological blood flow rates and wall shear stresses needed to activate mechanosensing/mechanotransduction pathways, thus, deviating from the realistic transport exchange mechanisms at the level of brain capillaries (25, 26). Although these systems are promising, they are limited in their ability to recreate a realistic and relevant BBB/NVU morphology.

As an alternative to simple and promising culture patterns, self‐assembled BBB/NVU models with ECM biomaterials have been developed to study organogenesis and perform permeability assays (16, 17, 18, 19). These models aim to capture the BBB/NVU morphology and development in terms of natural biological processes. However, such reported models lack adequate neural component (rather, they are focused on BBB features) (16, 19) or vascular networks (17, 18), and therefore, cannot reveal the direct interactions between the neural and vascular components of the NVU, failing to replicate the structures, function, and realistic three‐dimensional (3D) complexity of the NVU. In particular, the absence of a vascular system is detrimental to the inner cells in 3D culture systems, and 3D cultures under long‐term culturing consistently exhibit apoptotic cell death in the innermost regions (27, 28). Furthermore, differentiation of neuronal progenitors/neural stem cells (NSCs) is impaired without functional vasculature (29). Accurately replicating the interactions between the different neural cells and vascular component in the brain and adequately capturing the in vivo conditions are crucial for accurate NVU modeling.

To overcome the main limitations of the current NVU models, we present a method for reconstituting the in vitro 3D brain NVU with primary NSCs and brain microvascular endothelial cells (BMECs) of rats in the Matrigel ECM system, where all the cell types are spontaneously assembled into a modular organization reproducing the NVU structures (vascular and neural components), which are in dynamic and direct contact with each other. Confocal imaging and immunocytochemistry, transmission electron microscopy (TEM), electrophysiological recordings, permeability measurements, and protein expression analysis were used to quantitatively assess the NVU characteristics. Additionally, we investigated the pathological changes of the 3D NVU in the context of ischemia to evaluate its applicability to pathological study, employing a well‐characterized in vitro oxygen–glucose deprivation (OGD) pattern. Finally, we transplanted the 3D NVU to the infarcted side of cerebral ischemia rats to examine the functional effects of the grafted 3D NVU on the host ischemia brain.

## 2 MATERIALS AND METHODS

### 2.1 Animals usage

1‐day‐old, 10‐day‐old neonatal rats, and 2‐month‐old male Sprague‐Dawley (SD) rats (180–220 g) were purchased from the Experimental Animal Center, Chongqing Research Institute of Chinese Medicine (animal production number is SCXK 2019‐0014, Chongqing, China). Rats were maintained on in controlled conditions (22 ± 2°C, 12 h light/dark cycle, free access to food and water) in the Experimental Animal Center, the College of Pharmaceutical Sciences, Southwest University (Chongqing, China). The animal protocols were approved by the Southwest University Animal Use and Care Committee (animal approval number is SYXK (Yu) 2017‐0003). All animal experiments obeyed the ARRIVE guidelines and were carried out in accordance with the National Institutes of Health Guide for the Care and Use of Laboratory Animals (NIH Publication No. 8023, revised 1978).

### 2.2 Primary NSCs and BMECs culture

For all cell isolations, neonatal rats were anesthetized, disinfection by 75% ethanol, quickly decapitated and tissue of interest was collected. For isolation of hippocampal NSCs, the hippocampus from 1‐day‐old rats was removed and dissected in cold D‐Hanks balanced salt solution (without calcium and magnesium, GE Healthcare). The tissue was enzymatically digested using the ACCUTASE (Stem Cell) at 37°C for 5 min. The digested tissues were then washed with cold D‐Hanks and filtered through a 40 μm cell sieve followed by centrifuging at 150 × *g* for 5 min. For suspension cultures, the pellet was resuspended in approximately 7 ml DMEM/F12 medium (Gibco) supplement with 2% B27 (Gibco, without Vitamin A), 20 ng·ml^−1^ rhEGF (Stem Cell), 10 ng ml^−1^ rhbFGF (Stem Cell), 0.0002% heparin (Stem Cell), and cultured in T‐25 cm^2^ culture flasks (3–4 brains for one flask) in a 37°C, 5% CO_2_ incubator (Thermo Fisher 6000). A partial medium is changed (25–30% of total volume) after plating 2–3 days. NSCs were harvested for co‐culture when it formed to neurospheres with a diameter about 100–150 μm.

For BMECs culture (30), cortex tissue from 10‐day‐old rats were removed, and then, dissected to approximately 1.0 mm^3^ in cold D‐Hanks. Tissue was blown into homogenate using 25% bovine serum albumin (BSA) followed by centrifugation at 600 × g for 5 min. The microvessels obtained were enzymatically digested with 0.1% collagenase II (Sigma) at 37℃ for 5 min and the medium with 20% fetal bovine serum (FBS) was added to terminate the digestion. The digested tissue solution was centrifuged at 150 × g for 5 min, and then, seeded the obtained tissue pellet into T‐25 cm^2^ culture flasks (one brain for one flask) with approximately 4 ml DMEM/F12 medium supplemented with 20% FBS (Gibco), cultured in a 37°C, 5% CO_2_ incubator. The culture medium was changed every 2–3 days. BMECs were passaged at most three times before used in co‐culture.

### 2.3 Generation of 3D NVU

For co‐culture, medium contained neurospheres was centrifuged at 100 × *g* for 5 min and dissociated the obtained neurospheres to single cell using ACCUTASE. About 80%–90% confluent BMECs were trypsinized (with 0.02% EDTA) and centrifuged at 150 × g for 5 min and resuspension with serum‐free culture medium. NSCs and BMECs were mixed and resuspension with Matrigel (BD Biosciences, growth factor reduced) at a density ratio of 1:10. The final density of BMECs was 2.0 × 10^6^·ml^−1^, and the final density of NSCs was 2.0 × 10^5^·ml^−1^. The 100 μl cell/Matrigel mixture was then evenly spread on the bottom of the confocal dishes (NEST, 15 mm) or cell culture inserts (Greiner Bio‐One, 24‐well), and then, placed in an incubator to solidify. After 1 h, 50 μl mixture of NSCs/Matrigel was evenly spread on the surface of the solidified gels, which was further placed in the incubator for 2 h, and then, the culture medium was added. BMECs‐NSCs co‐culture system was grown in DMEM/F12: Neurobasal medium 1:1, 2% B27 (without vitamin A), 20 ng·ml^−1^ rh EGF, 10 ng ml^−1^ rh bFGF and the medium was supplemented with 1% FBS. After 3 days, B27 in the medium was replaced with B27 containing vitamin A. The BMEC‐NSC co‐cultures or control BMEC or NSC monocultures were analyzed following 3 or 7 days of culture, using live cell microscopy and immunofluorescence staining.

### 2.4 Immunochemistry

#### 2.4.1 3D cultures

As previous reported (31), the 3D cultures were washed once with Dulbecco's phosphate‐buffered saline (D‐PBS) and fixed with 4% paraformaldehyde overnight at room temperature. After washing with D‐PBS for 3 times × 10 min, the fixed cells were permeabilized by incubating with 200 µl of Tris‐buffered saline (TBS) buffer containing 0.1% (vol/vol) Tween‐20 (TBST) containing 0.5% (vol/vol) Triton X‐100 and 4% (vol/vol) goat IgG at room temperature for 1 h. The 3D cultures were blocked by incubating with a blocking solution containing 50 mM Tris (pH 7.4), 0.1% Tween‐20, 4% donkey serum, 1% BSA, 0.1% gelatin, and 0.3 M glycine at 4°C overnight with gentle rocking. After washing with TBST, the 3D cultures were incubated with primary antibodies in the blocking solution at 4°C for 24 . After washing five times with TBST, the cells were then incubated with TBST overnight by gentle rocking at 4°C, and then, further incubated with Alexa Fluor secondary antibodies (Abcam) for 5 h at room temperature with gentle rocking. To avoid fluorescence quenching, a drop of antifade glass with DAPI (Life Technologies) was added on top of the fixed/stained thin‐layer 3D cultures before imaging.

#### 2.4.2 Brain slices

On day 14, after 3D cultures transplantation, rats were deeply anesthetized with sodium pentobarbital and perfused transcardially with 4% paraformaldehyde. Brains were removed for paraffin embedding. The paraffin blocks were then cut into 5 μm sections (Leica SM2010R sliding microtome, Leica Microsystems Inc., Buffalo Grove, IL, USA), mounted on polylysine‐coated glass slides (Thermo Scientific), then, incubated at 45°C overnight. The sections were deparaffinized by two changes of xylene for 5 min each, and then, serially transferred to 100%, 90%, and 70% ethanol solution for 1 min each. The sections were then rinsed with distilled water for 5 min. For immunostaining, the antigen retrieval was performed by heating the slides for 30 min in Citrate‐EDTA Buffer containing 10 mM citric acid (pH 6.2), 2 mM EDTA and 0.05% Tween‐20. The brain sections were washed with 1 × Phosphate‐buffered saline (PBS) (3 × 10 min) and blocked in a blocking solution (0.3% Triton X‐100 in 1 × PBS+10% serum, which was generated from the species of the secondary antibodies) at room temperature for 1 h. The sections were then incubated in the primary antibody solution at 4°C overnight. After primary incubation, the brain sections were washed in PBS, followed by secondary antibody incubation for 2 h at room temperature. After a final wash step, the brain sections were mounted in fluorescence mounting medium with DAPI (Life Technologies) for image capture. All the used antibodies are listed in Table S1.

### 2.5 OGD and vascular endothelial growth factor (VEGF) or edaravone treatment for 3D cultures

For OGD, 3D cultures were placed in an anaerobic chamber (BINGDER150, Germany) containing a gas mixture of 5% CO_2_, 95% N_2_ at 37°C for 8 h. Normal culture media were replaced with deoxygenated, glucose‐free Earle’s balanced salt solution (EBSS, in mg/L: 6800 NaCl, 400 KCl, 264 CaCl_2_·2H_2_O, 200 MgCl_2_·7H_2_O, 2200 NaHCO_3_, 140 NaH_2_PO_4_·H_2_O, pH 7.2). Control 3D cultures were placed in EBSS containing 25 mM glucose and incubated under normal tissue culture conditions for the same period. 3D cultures were pretreated with VEGF (10 ng·ml^−1^) or edaravone (100 μM) for 16 h and SU1498 (10 μM) for 2 h before exposing to OGD and continue to treat with above reagents and incubate in OGD conditions for 8 h (Figure S1).

### 2.6 Western blot assay

Recovering cells from Matrigel Matrix of 3D cultures by using Cell Recovery Solution (Corning). The proteins in the 3D cultures were then collected according to the instructions of the manufacturer (CST). Samples from at least three independent experiments were harvested and analyzed in each group. To avoid protein degradation, RIPA lysis buffer supplement with protease inhibitor cocktail (HY‐K0010, HY‐K0022, HY‐K0023, MCE) was used. The VEGF‐treated and VEGF + SU1498‐treated 3D cultures were incubated in OGD conditions for 8 h, and then, the proteins were harvested for western blotting. All the used antibodies are listed in Table S2.

### 2.7 5‐ethynyl‐2’‐deoxyuridine (EDU) labeling proliferating cells of 3D cultures

To label the proliferating cells, EDU was added to the medium for 24 h and the final work concentration is 10 mM. The treated 3D cultures were then fixed with 4% paraformaldehyde. EDU labeling for S‐phase cells was detected using the BeyoClick™ (Beyotime), and cells were stained for nucleus with DAPI.

### 2.8 TEM analysis of tight junction

3D cultures were prefixed with 2.5% glutaraldehyde in 0.1 M PBS at 4°C, overnight, postfixed in 1% buffered osmium tetroxide, dehydrated in graded alcohols, embedded in Epon 812, sectioned with ultramicrotome (Leica UC7) and stained with uranyl acetate and lead citrate. The intercellular tight junction of the 3D NVU were observed with a transmission electron microscopy (Hitachi‐HT7700).

### 2.9 Electrophysiological recordings (32)

The 3D cultures were placed in oxygenated medium containing (in mM) 85 NaCl, 75 sucrose, 2.5 KCl, 25 glucose, 1.25 NaH_2_, and 24 NaHCO_3_PO_4_, 4 MgCl_2_ and kept in the same solution at room temperature until recording. We performed whole‐cell patch‐clamp recordings using artificial cerebrospinal fluid containing (in mM) 126 NaCl, 2.5 KCl, 26 NaHCO_3_ 2 CaCl_2_, 2 MgCl_2_, 1.25 NaH_2_PO_4_, and 10 glucose) gasses with 95% O2/5% CO2. Double patch Clamp 10 amplifier and digitizer system (Sutter Instrument) was used for recordings. The Clampfit 10 software (Molecular Devices) was used to analyze the data.

### 2.10 Cell viability assay

#### 2.10.1 Apoptotic cell assay

Apoptotic cell was determined using a Hoechst Staining Kit (Beyotime) containing Hoechst 33258.

#### 2.10.2 Live/Dead Assay

Cell viability was determined using a Live/Dead Viability Assay Kit (Bestbio) containing calcein AM, which stains live cells green, and PI, which stains dead cells red.

### 2.11 Cytokines assay

The contents of VEGF, angiopoietin‐1(ANG‐1), brain‐derived neurotrophic factor (BDNF), ciliary neurotrophic factor (CNTF), glial cell line‐derived neurotrophic factor (GDNF), nerve growth factor (NGF), and neurotrophin‐3(NT‐3) in the supernatant was detected by an enzyme‐linked immunosorbent assay (ELISA) according to the manufacturer’s instructions (Sino best biological technology), respectively. Absorbance was measured at 490 nm using a microplate ELISA reader (BioTek, Winooski, VT, USA). Each final value was quantified against a standard curve calibrated with known amounts of protein.

### 2.12 Permeability measurement of 3D structures

#### 2.12.1 FITC‐Dextran

Solutions of 70 KDa FITC‐Dextran (Sigma‐Aldrich) were prepared at 100 μg·ml^−1^ in cell culture media. Then, the vascular compartment of the NVU in dishes was incubated with FITC‐Dextran solution for 2 h. At the 2 h mark, the 3D cultures were washed three times with wash buffer (100 ml PBS, 0.95 g MaCl_2,_ 0.11 g CaCl_2, and_ 2 g BSA). For observation of the integrity of the vascular network, an inverted fluorescence microscope (Leica DFC310 FX) was used.

#### 2.12.2 Sodium Fluorescein

Briefly, the 3D cultures were generated in the cell cultures inserts and the culture medium in apical chamber of the inserts was replaced with 0.5 ml DMEM‐F12 medium containing 100 μg·ml^−1^ sodium fluorescein. After 2 h incubation, the samples were taken from the basolateral chamber, and the absorbance of the samples were measured by plate reader.

### 2.13 3D cultures transplanted into cerebral ischemic rats

#### 2.13.1 Rat focal cerebral ischemic models, neurological deficiency

Male Sprague‐Dawley rats, 180–220 g, were used to establish the middle cerebral artery occlusion (MCAO)‐induced cerebral ischemic models via electrocoagulation according to reported protocol (33, 34). Briefly, SD rats were anesthetized with isoflurane (2–3% in oxygen) and placed into a stereotaxic frame. A longitudinal incision was made at the junction of the temporalis muscle and parietal bone. The temporalis muscle was then isolated from the harnpan using the hilt to expose the temporal fossa, and a small hole was made using a small electric drill and extended in the temporal fossa. After exposed the middle cerebral artery (MCA), bipolar coagulation forceps were used to coagulate MCA downward at the intersection of MCA and inferior vena cava. the wound was filled with hemostatic sponge, and the muscle and skin were sutured, respectively, after the operation. After 2 h from rats awakening, the modified neurological severity scores (mNSS) was used to screen for successful MCAO rats, as previously reported (35, 36). The MCAO was considered successful in the rats when the mNSS scores was between 4 and 12.

#### 2.13.2 Transplantation of 3D cultures

For transplantation of 3D cultures, the skin of successful MCAO rats was cleaned with alternating applications of alcohol three times and the infarction side was exposed again. The 3D cultures were then placed onto the raw edges of occlusive MCA. The wound was filled with hemostatic sponge, and the muscle and skin were sutured, respectively, after the operation. All postoperative animals were observed daily for 14 days to monitor recovery, behavior, and incision healing. The rats were injected daily with Cyclosporine A (i.p., 10 mg·kg^−1^, MCE) for immunosuppression from day 1 to day 14 after transplantation. On day 14, the rats were transcardially perfused with heparinized saline (0.9%) and the brain was removed and fixed with 4% paraformaldehyde.

### 2.14 5‐Triphenyte‐trazoliumchloride (TTC) staining for brain slices

On day 14 after 3D cultures transplantation, rats were deeply anesthetized with sodium pentobarbital and the animals were decapitated. The brains were removed and briefly cooled with a temperature about −80°C. The brain was sliced coronally in a 2 mm interval with a brain matrix, total seven coronal sections, for infarct volume assessment. The sliced sections were then subsequently stained with 1% TTC (Sigma‐Aldrich) at 37°C for 30 min. Finally, the stained sections were transferred into 4% paraformaldehyde at 4°C for fixing. Image‐Pro Plus (Version 6.0., Mediea Cybernetics, Inc.) was used to calculate infarct size. Infarct size = The size of left hemisphere − (The right hemisphere − Measured infarct size).

### 2.15 Image acquisition and analysis

The 3D reconstructions and cross‐sections of the 3D cultures were captured by using a confocal microscope (NIKON, A1 + R10802; OLYMPUS, FV1200). Images were analyzed by Image J (NIH) and NIKON Nd2 plugin was used. The brain sections were captured under a 20‐fold magnification using a microscope equipped with a PL FLUOTAR 20x/0.4 objective and a Leica DFC310 FX Digital camera. For each technical replicate, five images were taken from different areas on the coverslip prior to calculating a mean value of cell counts for each sample. All parameters, such as exposure time, gain, brightness, and contrast, of each image were maintained the same for each fluorescence channel.

### 2.16 Statistical analysis

All experiments consisted of three or six biological replicates, each consisting of three technical replicates. Statistical analysis was performed using SPSS 20 (IBM) and graphs were drawn in Prism 8 (Graph Pad). Normality was assessed by the Shapiro–Wilk test. Statistical differences between two groups were analyzed with two‐tailed, unpaired Student’s t‐tests. ANOVA combined with Holm‐Sidak or Tukey's multiple comparison test was used when data from multiple groups were analyzed. Data are presented as the mean ± SD. A *p* value of less than 0.05 was considered to be statistically significant.

## 3 RESULTS

### 3.1 Generation of 3D NVU models with primary NSCs and BMECs

**FIGURE 1:**
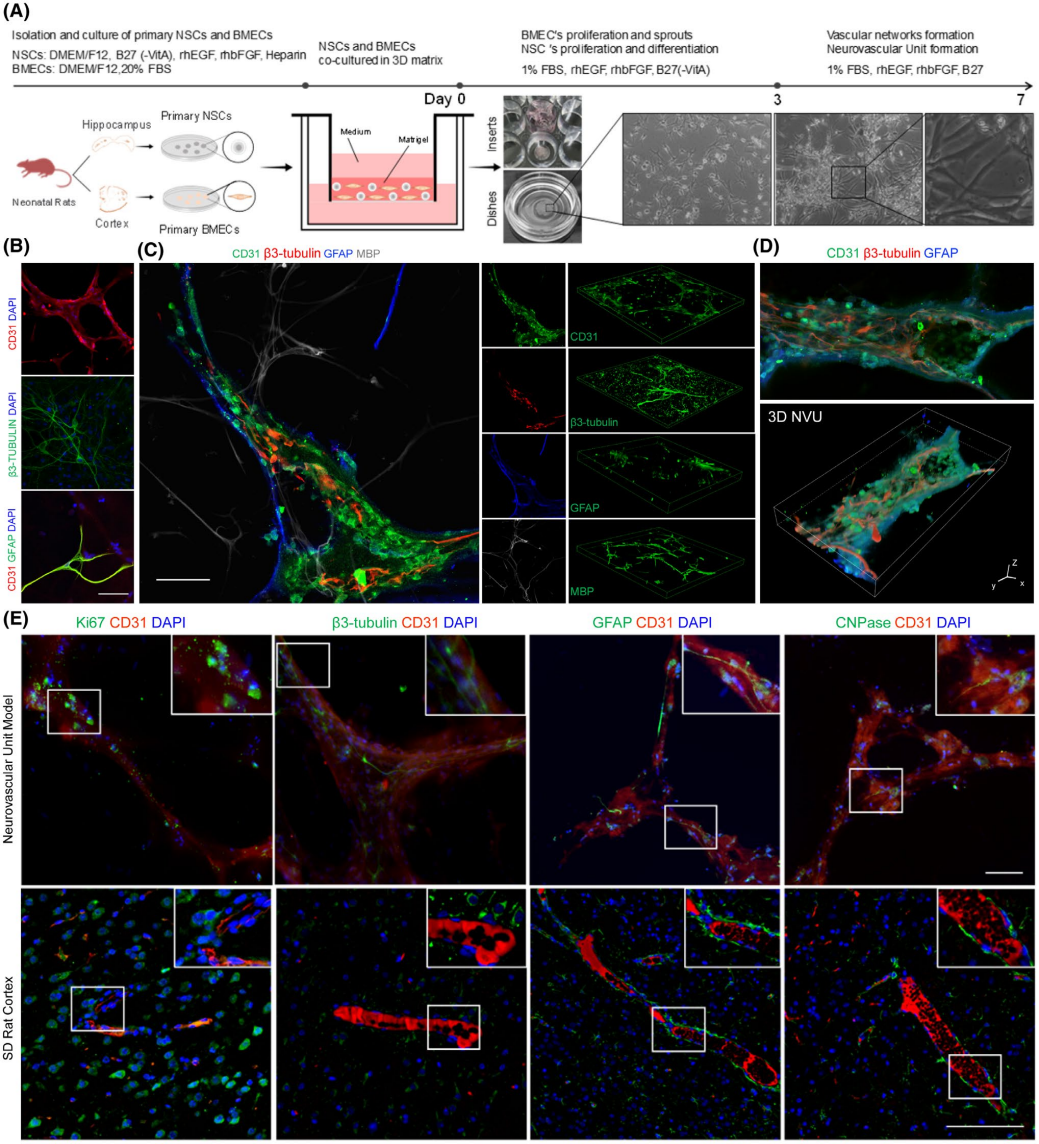
Generation of 3D NVU model from primary NSCs and BMECs. (A) Schematic of the protocol used for the differentiation of NSCs and BMECs into the 3D NVU. (B) Representative immunofluorescence of CD31‐expressing BMECs, β3‐tubulin‐expressing neurons, and the contact between BMECs and GFAP‐expressing astrocytes in the 3D NVU (bar = 100 μm). (C) Relative space position relations between vascular‐like structures and the differentiation of the NSCs, neuron (β3‐tubulin), astrocyte (GFAP), oligodendrocyte (MBP), and vessel (CD31), bar = 100 μm. (D) Representative 3D reconstruction of the NVU model (bar = 10 μm). (E) Relative space position relations between the neural component and the vascular component in the cortex or 3D NVU. This 3D NVU model is highly similar to the complex structures of the NVU in vivo. The top panel shows the 3D NVU model, and the bottom panel shows the cortex of SD rats, neuron (β3‐tubulin), astrocyte (GFAP), oligodendrocyte (CNPase), vessel (CD31), and dividing cell (Ki67). The bar represents 100 μm

With the aim of reconstituting a 3D NVU with vascular‐like structures and neural component (neuron, glia) in vitro—similar to an NVU in vivo—we used the primary NSCs and BMECs from rats (Figure S2) to develop a multistep protocol for modulating the formation of neural component and vascular structures through programming of the internal interactions and internal tissue mechanics, which is called self‐organization (Figure 1A). Confocal imaging revealed the formation of complex, interconnected networks of CD31^+^ endothelial tubes and β3‐tubulin^+^ neurons, as well as close direct contact between vascular‐like structures and astrocytes (Figure 1B). In this self‐organizing NVU model, BMEC‐derived vascular networks formed vascular component was entwined neural component, including neurons, astrocytes, oligodendrocytes differentiated from NSCs and exhibit 3D structures, respectively (Figure 1C). 3D reconstruction showed that the NSCs differentiated neurons and astrocytes deeper in the contact or wrap vascular structures, together formed the holism unit (Figure 1D, Figures S3 and S4).

Moreover, the relative space position relations between the vascular and neural components of the 3D NVU model were similar to those for the NVU in the cortex of rats, as vessels from the cortex or vascular‐like structures from the 3D NVU were partially ensheathed by neurons, astrocytes, and oligodendrocytes. Through co‐staining of the neuron marker β3‐tubulin, astrocyte marker GFAP, or oligodendrocyte marker CNPase with the BEMC marker CD31 (separately), the neural component in the 3D NVU surrounded by vascular‐like structures was identified (Figure 1E). Strikingly, there were vast dividing cells (Ki67^+^) adjacent to the NVU planar vascular plexus, both in the 3D NVU and the rat cortex, which is also a prominent feature of interactions between vascular and neural component in NVU (Figure 1E). This 3D NVU not only exhibited vascular‐like phenotypes and the expression of endothelial markers, but also contained distinct neural component from recently reported BBB or NVU models (10, 11, 12, 15, 16, 18, 20, 37). The results indicate that our 3D NVU model is similar to the complex structures of the NVU in vivo.

### 3.2 Characteristics of vascular‐like structures in 3D NVU

**FIGURE 2:**
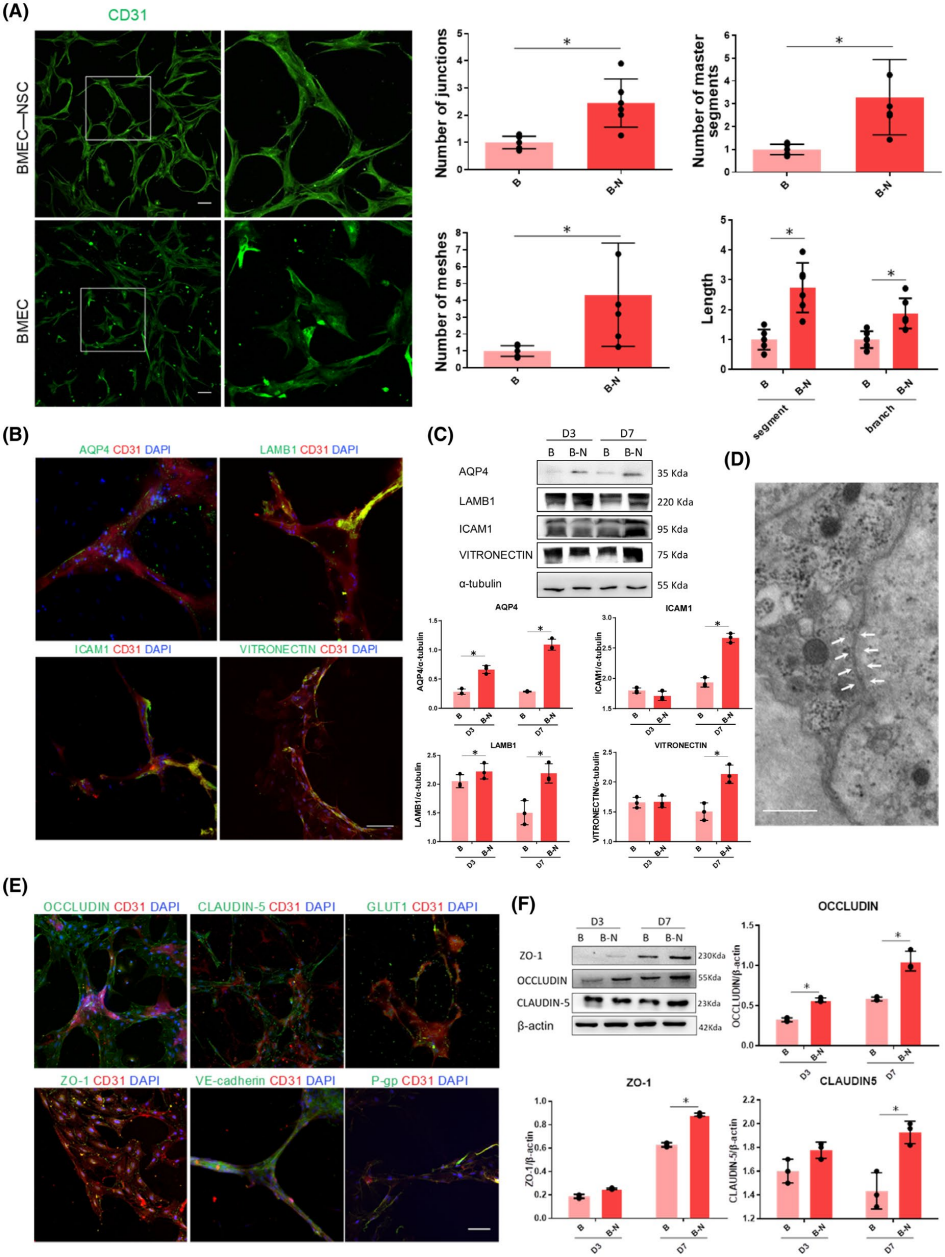
Comparison of the formation of BMEC‐derived vascular‐like structures in control BMEC cultures and BMEC‐NSC cultures. (A) Upper left, immunostaining for CD31 in the sectioned 3D NVU on day 7; bottom left, immunostaining for CD31 in BMEC cultures on day 7. The right panel shows a magnified view of the white box (bar = 50 μm; data are presented as mean ± SD, n = 6, ^*^ *p* < 0.05). (B) Cytoarchitectural characterization of vascular‐like structure phenotype. Immunostaining of endfeet between astrocytes and endothelium, as well as vascular markers on BMECs (bar = 100 μm). (C) Comparison of vascular structure‐related protein expression levels in control BMEC cultures and BMEC‐NSC cultures (data are presented as mean ± S.D, n = 3, ^*^ *p* < 0.05). (D) TEM morphological observations of tight junctions of the BBB in the 3D NVU (bar = 1 μm). (E) BBB‐like characterization of 3D NVU. Immunostaining of tight junction and adherens junction markers of the BBB in the 3D NVU (bar = 100 μm). (F) Comparison of tight junction protein expression levels in control BMEC cultures and BMEC‐NSC cultures (data are presented as mean ± SD, n = 3, ^*^ *p* < 0.05)

We investigated the transformation of the endothelial cells into vascular‐like networks. Whole‐mount immunostaining indicated that CD31^+^ endothelial tubes were formed in the 3D NVU on day 7, while control BMEC cultures lacked the formation of these whole tubes. Moreover, the 3D NVU contained more complex and complete networks of BMEC‐derived CD31^+^ vascular‐like structures than the BMEC cultures (Figure 2A, left). Quantification of the vascularization via ImageJ angiogenesis plug indicated that the 3D NVU had significantly more junctions, meshes, and master segments and longer vascular‐like structures than the BMEC cultures (Figure 2A, right). The results indicate that NSCs promoted the formation of vascular‐like structures.

During adulthood, bidirectional signaling between astrocyte endfeet and brain endothelial cells helps maintain vascular integrity (38). The indirect contact between vascular structures and astrocytes is a critical characteristic in the BBB of the NVU (39). The endfeet of astrocytes were visualized via immunostaining for Aquaporin‐4 (AQP4), which is a water channel with abundant astrocyte endfeet at the BBB (Figure 2B). Additionally, the vascular‐like structures were ensheathed by vascular basal proteins, such as LAMB1, ICAM1, and VITRONECTIN (Figure 2B). The expression levels of AQP‐4, LAMB1, ICAM1, and VITRONECTIN were increased significantly compared with those for the BMEC cultures (Figure 2C).

A critical characteristic of the BBB is a junction barrier formed by connected endothelial cells (5, 40). In addition to the expression of endothelial cell markers (Figure 2B), the 3D NVU exhibited tight junction markers (ZO1, OCCLUDIN, and CLAUDIN‐5), adherens junction marker (VE‐cadherin), glucose transporter (GULTI), and P‐glycoprotein (P‐gp) in the vascular‐like structures on day 7 (Figure 2E). Both the immunostaining and TEM results indicated that tight junctions were clearly observed and widely distributed in the vascular‐like structures. (Figure 2D,E). Additionally, the junctional protein expression levels in the 3D NVU were significantly increased compared with those for the BMEC cultures (Figure 2F). These results indicate that the NSCs facilitated the formation of functional endothelial junctions, leading to BBB‐like characteristics. NSC and BMEC co‐culturing gave rise to the distinct expression of junction markers, astrocytic endfeet, and basement membrane proteins, mimicking a BBB‐like phenotype; however, the structure differed from that of the naturally formed BBB. Thus, our 3D NVU not only exhibited vascular‐like phenotypes and the expression of endothelial markers, but also contained tight/adherens junctions and exhibited BBB‐like characteristics comparable to those of previously reported BBB models (12, 15, 37).

### 3.3 Characterizations of neural component in 3D NVU

**FIGURE 3:**
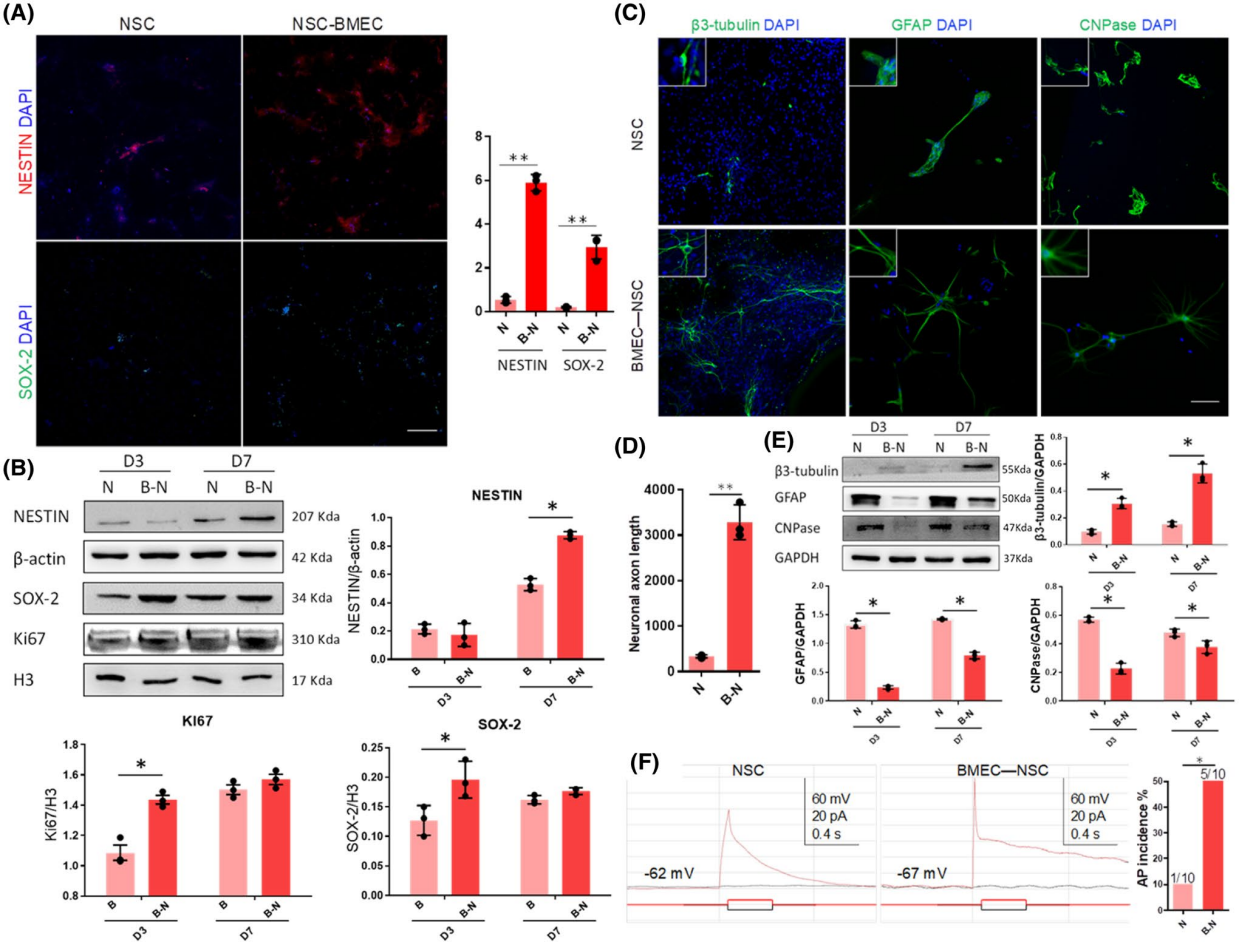
Proliferation and differentiation of NSCs in co‐culture or monoculture. (A) Immunostaining of NSCs markers in 3D NVU, bar = 100 μm, (Data are presented as mean ± SD, n = 3, ^**^ *p* < 0.01). (B) The comparison of NSCs marker expression levels in control NSC cultures and in BMEC‐NSC cultures (data are presented as mean ± SD, n = 3, ^*^ *p* < 0.05). (C) Immunostaining of neurons (β3‐tubulin), astrocytes (GFAP), and oligodendrocytes (CNPase) in 3D NVU, bar = 100 μm. (D) The comparison of axon length in control NSC cultures and in BMEC‐NSC cultures (data are presented as mean ± SD, n = 3, ^**^ *p* < 0.01). (E) Comparison of neuron, astrocyte, and oligodendrocyte marker expression levels in control NSC cultures and BMEC‐NSC cultures (data are presented as mean ± SD, n = 3, ^*^ *p* < 0.05). (F) Voltage traces of current clamp recordings of a cell in control NSC cultures and BMEC‐NSC cultures on day 7 in response to hyperpolarizing (–10 pA) and depolarizing (+10 pA) current steps (data are presented as mean ± SD, n = 10, ^*^ *p* < 0.05)

Previous studies indicated that the proliferation and differentiation of NSCs are induced by factors secreted by endothelial cells in an indirect co‐culture system (29). What are the proliferation and differentiation of NSCs in a 3D direct co‐culture system?It was observed that the number of NESTIN^+^ and SOX‐2^+^ cells was significantly increased compared with that for NSC cultures (Figure 3A). Additionally, western blotting results indicated that the expressions of specific marker proteins NESTIN and SOX‐2 were significantly upregulated (Figure 3B). These results indicated that the BMECs promoted the proliferation of NSCs during the direct co‐culturing.

Regarding the differentiation of NSCs, after the co‐culturing, the three types of nerve cells derived from the co‐cultured NSCs all had a more mature cell morphology (TUJ1^+^ axon, GFAP^+^ intermediate filament, and CNPase^+^ cell body) than the NSC cultures (Figure 3C). Specifically, neurons differentiated from NSCs in the 3D NVU exhibited reduced dendritic complexity, and the axon length was increased significantly (Figure 3D). GABAergic neurons and dopaminergic neurons also existed in the 3D NVU (Figure S5). The proportion of neurons that were differentiated increased, while the proportion of astrocytes and oligodendrocytes that were differentiated decreased (Figure 3E). These results indicate that in the reconstituted 3D NVU model, BMECs successfully promoted NSC self‐renewal, colony formation, and differentiation into neurons, promoting the morphological maturity of the NSCs and impeding their differentiation into glial cells.

The formation of vessels is critical for the maturation of cortical neurons (41). Thus, we performed whole‐cell patch‐clamp recordings to characterize the neuronal activity in NSC and BMEC‐NSC cultures. Five of 10 cells randomly selected from the NSC‐BMEC cultures produced action potentials with spike frequency adaptation, whereas the remaining five cells did not produce action potentials (Figure 3F). In contrast, only one of 10 cells from the control NSC culture produced single low‐amplitude action potentials while the remaining nine cells did not produce any action potentials. The incidence rate of obtaining neurons that were able to produce action potentials was significantly higher in BMEC‐NSC cultures compared to that in control NSC cultures (Figure 3F).

### 3.4 Cell viability in 3D NVU

**FIGURE 4:**
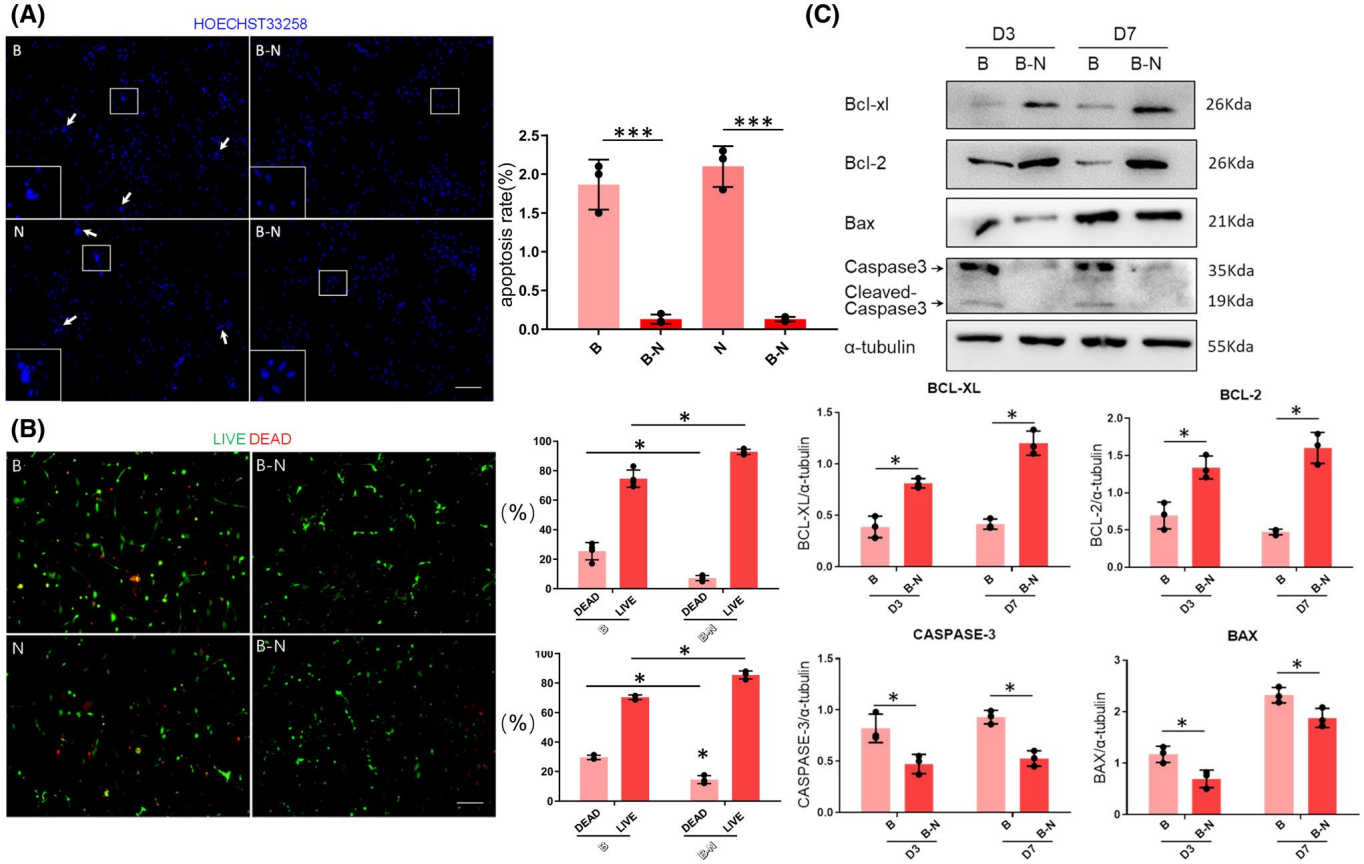
Comparison of cell viability in monoculture and co‐culture. (A) Apoptosis rates for control NSC and BMEC cultures and BMEC‐NSC cultures (data are presented as mean ± SD, n = 3, ^***^ *p* < 0.001). (B) Numbers of live/dead cells for control NSC or BMEC cultures and BMEC‐NSC cultures (data are presented as mean ± SD, n = 3, ^*^ *p* < 0.05). (C) Western blot analysis of apoptosis‐related proteins in the control NSC and BMEC cultures, as well as the BMEC‐NSC cultures (data are presented as mean ± SD, n = 3, ^*^ *p* < 0.05)

When cultured in Matrigel for 7 days (supplemented with a low‐serum medium), the staining of Hoechst 33258 and live/dead cells indicated that the control NSC and BMEC cultures exhibited significantly higher apoptotic signals and significantly higher cell death rates than the 3D NVU (BMEC‐NSC cultures) (Figure 4A,B). Additionally, compared with control NSC and BMEC cultures, in the 3D co‐culture system, the expressions of the apoptosis‐promoting proteins Bax and Caspase3 were significantly reduced, and the expression levels of the apoptosis‐inhibiting proteins Bcl‐xl and Bcl‐2 were significantly increased (Figure 4C). The results indicate that in the co‐culture, the NSCs and BMECs maintained cell–cell interactions through secreted factors or direct contact and inhibited the cell apoptosis and death. This provided evidence that NSCs and BMECs still have the characteristics of coordination and mutual support when co‐cultured in vitro and can interact to form an NVU.

### 3.5 Paracrine signaling via soluble factors in 3D NVU

**FIGURE 5:**
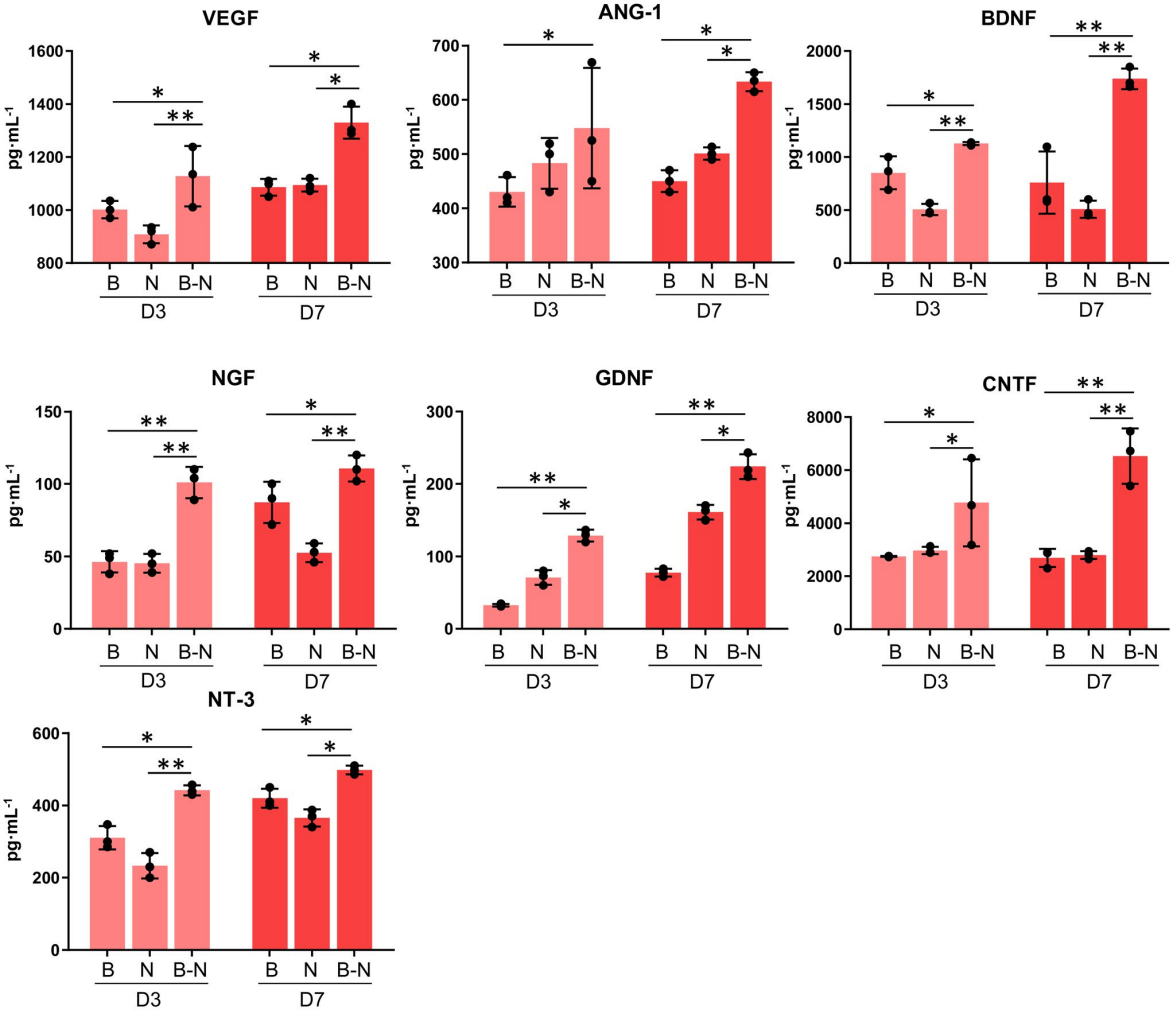
Comparison of soluble factor expression in co‐culture and monoculture. The growth factors were detected via an ELISA for the control BMEC and NSC cultures and the BMEC‐NSC cultures on days 3 and 7 (data are presented as mean ± SD, n = 3, ^*^ *p* < 0.05, ^**^ *p* < 0.01)

Accumulating evidence indicates that the mutual signal conduction between NSCs and endothelial cells can simultaneously regulate the processes of neurogenesis and angiogenesis in the brain (42). ELISA was performed to detect the changes in the related factors for the control NSC or BMEC cultures and the BMEC‐NSC cultures. The results showed that compared with the control NSC and BMEC cultures, the expression levels of the angiogenesis‐related soluble factors (VEGF and ANG‐1) and neuroprotection soluble factors (BDNF, NGF, GDNF, CNTF, and NT3) were significantly increased for the BMEC‐NSC cultures (Figure 5). These results indicate that NSCs and BMECs can coordinate with each other and promote the expression of soluble factors, maintaining the homeostasis of the NVU.

### 3.6 OGD‐damaged 3D NVU exhibited pathology characteristics of ischemic stroke, whereas VEGF or edaravone had protective effect

**FIGURE 6:**
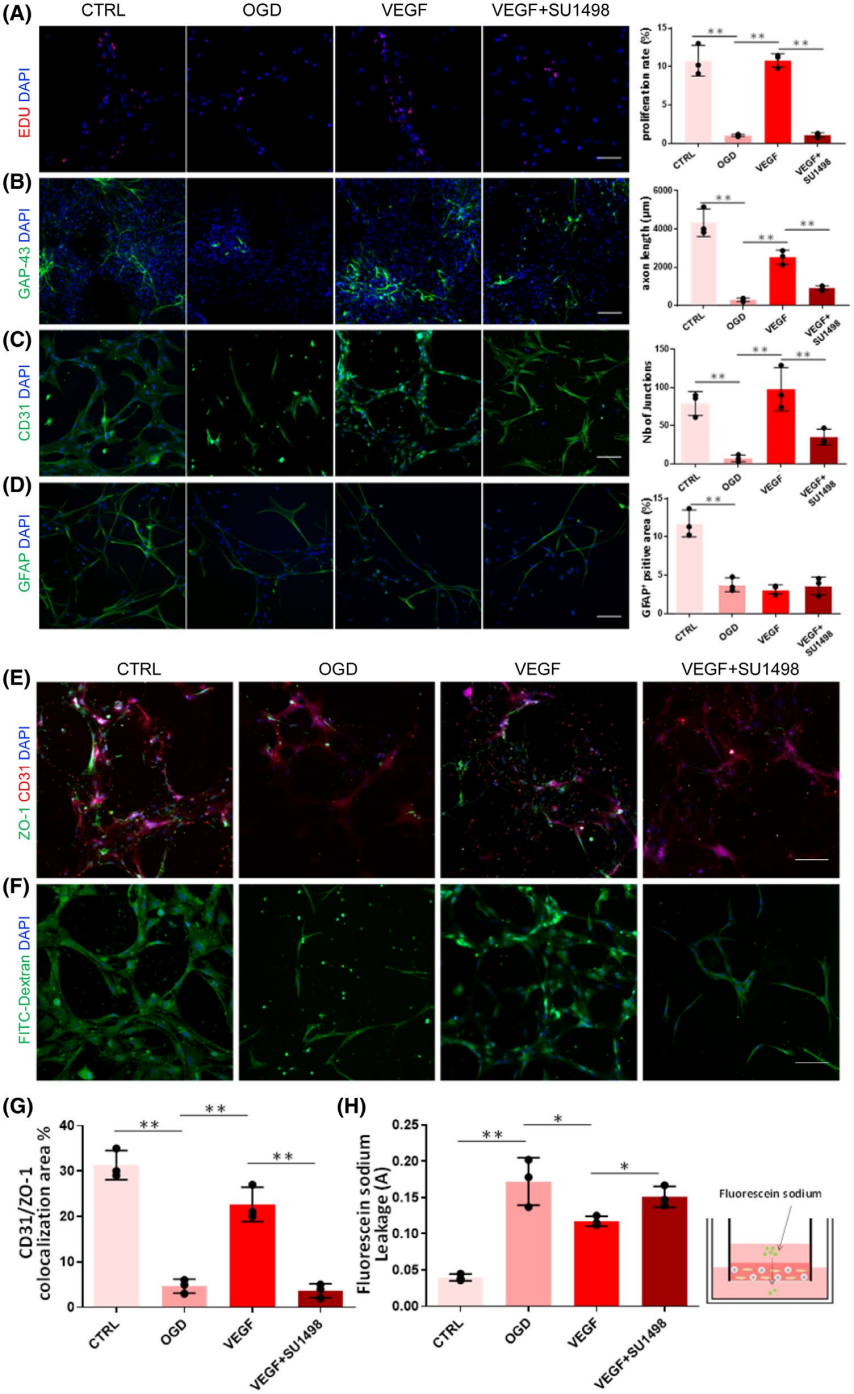
Protective effects of VEGF on the OGD‐damaged 3D NVU. a. Effect of VEGF on the proliferation of the OGD 3D NVU (data are presented as mean ± SD, n = 3, ^**^ *p* < 0.01). (B) Effect of VEGF on the axon length of neurons in the 3D NVU (data are presented as mean ± SD, n = 3, ^**^ *p* < 0.01). (C) Effect of VEGF on the vascular‐like structure phenotype of the 3D NVU (data are presented as mean ± SD, n = 3, ^**^ *p* < 0.01). (D) Effect of VEGF on the astrocyte microenvironment in the 3D NVU (data are presented as mean ± SD, n = 3, ^**^ *p* < 0.01). (E) Immunostaining of tight junction in 3D NVU, bar = 100 μm. (F) FITC‐dextran diffusion in the 3D NVU, bar = 100 μm. (G) Measurement of CD31/ZO‐1 colocalization in the 3D NVU (data are presented as mean ± SD, n = 3, ^**^ *p* < 0.01). (H) Measurement of sodium fluorescein leakage in the 3D NVU (data are presented as mean ± SD, n = 3, ^*^ *p* < 0.05, ^**^ *p* < 0.01)

In addition to the physical features, we evaluated the biological function, pathological and pharmacological study applicability of the 3D NVU. We developed an OGD model of the 3D NVU to model the ischemic stroke by using an oxygen‐ and glucose‐free medium and an oxygen‐free chamber (Figure S1). We then tested the effects of VEGF on the vascular and neural components of the OGD‐damaged 3D NVU. VEGF, which is secreted by endothelial cells, can stimulate angiogenesis and neurogenesis (43). After the 3D NVU was exposed on the OGD, in contrast to the control (CTRL) group, the cell proliferation rate was significantly reduced, indicating that the cell viability of the 3D NVU was inhibited. However, VEGF promoted the cell proliferation in the 3D NVU, and this effect was inhibited by specific inhibitors for VEGF/VEGFR2 (SU1498, 10 mM), which suggests that the pathological damage of the 3D NVU was reversed (Figure 6A). Compared with the CTRL group, the length of the neuronal axon of the 3D NVU under OGD was significantly reduced. However, after the VEGF treatment, the length of the neuronal axons increased significantly. This effect was inhibited by SU1498 (Figure 6B). We further observed the effect of the VEGF on damaged vascular‐like structures. After the OGD injury, the vascular‐like structures were disaggregated, and the junction number was significantly reduced. However, the VEGF maintained the integrity of the vascular‐like structures, and the number of junctions was significantly increased. This effect was also inhibited by SU1498 (Figure 6C). Astrocytes are an important part of the NVU of the brain. Compared with the CTRL group, the number of astrocytes in the 3D NVU was significantly reduced after the OGD (Figure 6D), but the VEGF had no effect on this pathological state.

We further examined the BBB function of 3D NVU under OGD. To measure tight junction integrity of 3D NVU under OGD, we performed immunostaining of ZO‐1. ZO‐1^+^ junction was mainly distributed, arranged in a continuous and close manner without obvious gap in the vascular‐like structures in CTRL group (Figure 6E). However, the expression of ZO‐1 was decreased and the annular structure was broken or even disintegrated in the OGD group. Compared with the OGD group, the colocalization of CD31/ZO‐1 was increased significantly and the junction phenotype was improved by VEGF but the effect was inhibited by SU1498 (Figure 6E,G). The permeability of the vascular structures in the 3D NVU was investigated using the FITC‐dextran assay and the sodium fluorescein assay. The OGD conditions led to decreased FITC‐dextran filling and increased sodium fluorescein leakage, whereas VEGF increased the FITC‐dextran distribution and reduced sodium fluorescein leakage (Figure 6F,H).

To further verify the pharmacological study availability of 3D NVU, a strong free radical scavenger for cerebral ischemia was employed. In contrast to the OGD group, edaravone increased the cell survival (Figure S6a) and repressed the expression of ROS and superoxide anion (Figure S6b,c). Also, edaravone promoted the expression of SOD, suppressed the production of NO and MDA, and reduced the leakage of LDH (Figure S6d–g), indicted that the oxidative stress damages of OGD exposed 3D NVU was improved by edaravone. The cell apoptosis in 3D NVU was repressed by edaravone which is proved by the reduced apoptosis cells(Figure S6h), increased mitochondrial membrane potential(Figure S6i), increased Bcl‐xl and Bcl‐2 expression, and decreased Bax and caspase 3 expression in 3D NVU of edaravone group, compared with that in OGD group(Figure S6j). Collectively, these results indicated that the 3D NVU provides a platform for performing pathological and pharmacological study, as the OGD exposed 3D NVU exhibited pathological characteristics of cerebral ischemia, which can be reversed by effective clinic drugs, like edaravone.

### 3.7 Transplanted 3D NVU promoted angiogenesis and neurogenesis in ischemic brain of MCAO rats

**FIGURE 7:**
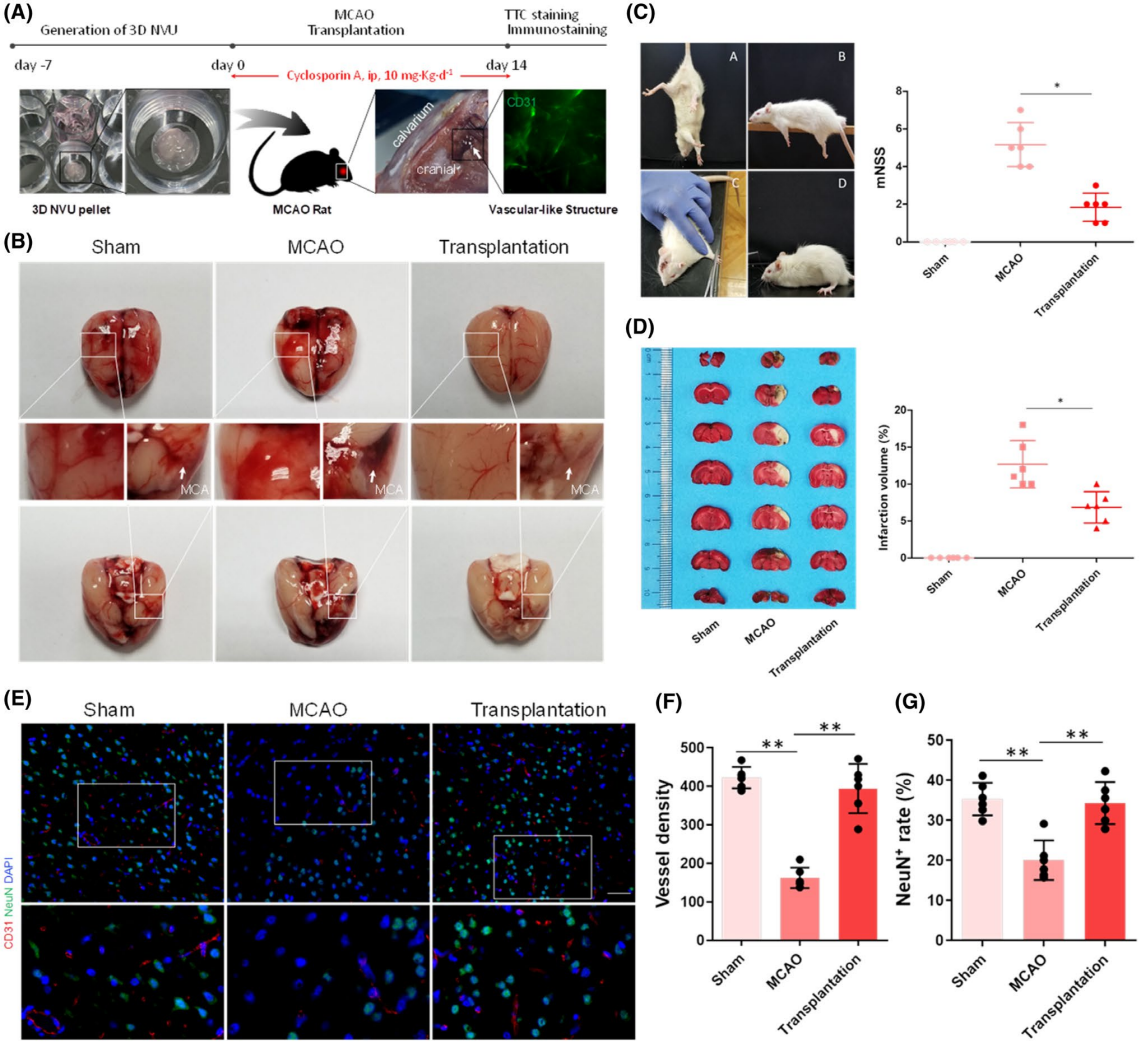
Transplantation of 3D NVU into the infarcted side of cerebral ischemia rats. (A) Process sketch of the 3D NVU transplantation. (B) Morphology of the cerebral infarction lateral surface before and after transplantation. (C) mNSS scores of ischemic rats: A, tail suspension.; B, balance beam tests; C, sensory tests; D, reflex absence and abnormal movements and walk tests (data are presented as mean ± SD, n = 6, ^*^ *p* < 0.05). (D) TTC staining for the infarcted brain volume before and after transplantation (data are presented as mean ± SD, n = 6, ^*^ *p* < 0.05). (E–G) The vessel and neuron densities (CD31^+^/NeuN^+^) were determined via paraffin section immunofluorescence staining (data are presented as mean ± SD, n = 6, ^**^ *p* < 0.01)

To determine whether the 3D NVU exhibits in vivo functional activity, we implanted it into the infarct area of immune‐inhibited rats (cyclosporine A, i.p, 10 mg·kg·d^−1^) and subsequently performed mNSS and TTC staining. For the infarction area, the transplanted 3D NVU still exhibited CD31^+^ vascular‐like structures on day 14 after transplantation, indicating that the activity of the 3D NVU that we constructed was maintained in vivo (Figure 7A). The infarction tissue in the MCAO group was liquefied on day 14, whereas the cerebral tissue morphology of the infarction area was maintained after 3D NVU transplantation (Figure 7B). Additionally, the mNSS scores of the Transplantation rats were reduced after the transplantation of the 3D NVU, compared with the MCAO group (Figure 7C). TTC staining results indicated that the infarct volume of cerebral ischemic rats transplanted with the 3D NVU was significantly reduced compared with the MCAO group (Figure 7D). Additionally, the immunostaining results indicated that the blood vessel density and neuron density on the infarction side were significantly higher for the transplantation group than for the MCAO group (Figure 7E–G). These results indicate that the 3D NVU may reduce the cerebral infarction volume of ischemic rats by promoting angiogenesis and neurogenesis in the infarcted area, suggesting that the 3D NVU is functional in vivo.

## 4 DISCUSSION

The NVU combines neurons, vessels, and ECM into a unique structural and functional unit, and its homeostasis is essential for maintaining brain health (2, 44, 45). Because direct research on the NVU is limited, modeling the NVU with vascular structures is critical for basic research and drug screening for related diseases. The discovery of NSCs and their microenvironment has led to new strategies for the investigation of neurogenesis and angiogenesis in most neurodegenerative diseases and NVU modeling. Previous studies have indicated that there is a causal relationship between angiogenesis and neurogenesis in the brains of adult songbirds; they coordinate and promote each other (46). Endothelial cells promote the neurogenesis of embryonic and adult NSCs/progenitor cells (29, 47), while NSCs/progenitor cells promote the differentiation and angiogenesis of endothelial cells (44, 48). Because of the significant interactions between NSCs and endothelial cells, the integration of neurogenesis and angiogenesis is a promising field for NVU modeling and is currently providing us with powerful tools to deepen our understanding of the brain.

Although current ambitious and promising projects aim to replicate human BBB/NVU “on a chip” or “in biomaterial” in vitro by co‐culturing endothelial cells with pericytes, astrocytes, neurons, or other types of cells (10, 11, 14, 16, 18, 20, 49), these systems do not adequately reflect the realistic complex 3D environment and the relevant NVU morphology in vivo, with the exception of explore the neurovascular interactions (50). Generally, 3D co‐culture environments are used to mimic the architecture of native tissues. The presented 3D NVU co‐culture systems with biomaterials similar to those of the ECM are formed by programming mature cell–cell and neural component/vascular component interactions on the basis of natural biological processes, which is called self‐organization. The cells spontaneously assemble into ordered structures and do not require preformed patterns. This 3D NVU realistically reconstituted the 3D complexity of the NVU, with neural component, vascular component, and BBB‐like characteristics.

The design of a co‐culture system often aims to capture cellular interactions. These interactions can occur via direct cell–cell contact, cell–ECM adhesion, or transfer of signaling molecules. The types of interactions that occur in a co‐culture system significantly affect the outcome of the system. In the present study, we successfully reconstituted a 3D NVU model including vascular and neural components by using the close relationships between NSCs and endothelial cells. In this 3D NVU, the vascular structures formed by BMECs and the neural component differentiated from NSCs are self‐assembled with the support of the matrix as an orderly interaction structure, through cell–cell adhesion, cell–ECM adhesion, and paracrine signaling with soluble factors. Paracrine signaling is important for regulating the behavior of stem cells and terminally differentiated cells within a co‐culture (51). Additionally, paracrine signaling through soluble factors is critical to BMEC–NSC interactions in a 3D co‐culture system. In a co‐culture system, the differentiation of stem cells is dependent on the origin of the terminally differentiated assisting cells and the matrix biochemistry. Additionally, the secretion of soluble factors by stem cells affects the behaviors of terminally differentiated cells (52, 53). For tissue engineering applications, it is important to consider that the cell interactions in co‐cultures are seldom unidirectional; typically, both cell populations are affected. Our data suggested that cell interactions from the 3D co‐culture local environment promoted the transformation of the BMECs into vascular‐like structures and promoted NSC differentiation into neurons (Figures 2 and 3). Compared with the control NSC and BMEC cultures, the 3D co‐culture system formed a more complete vascular‐like structures and neural networks.

Additionally, primary cultured NSCs and BMECs are used in this 3D NVU, because immortal cells tend to have less functionality than mortal cells, do not always express the same transporters and tight junction proteins as in vivo, and generally exhibit poor barrier function (54, 55). In BBB models, primary cells have exhibited higher functional output results than immortalized cell lines (56) and may better reflect the in vivo situation. Certainly, for therapeutic applications, it is important to verify that the neurovascular interactions identified in rodents also occur in humans. Clearly, there are differences between rodent models and humans. However, neurogenesis, angiogenesis, and their interactions occurs in the subventricular and subgranular zones of both adult humans and rodents; thus, it is practicable to investigate human neurovascular diseases using primary rodent cell‐derived NVU models. A key limitation in direct studying humans is access to living material. In previous BBB/NVU modeling studies, human‐induced pluripotent stem cell (iPSC)‐derived cells were widely employed to address this limitation (11, 12, 13, 57). The application of iPSC technology to model natural systems is satisfactory, in many aspects, though programming errors and genomic instability defects bring limitations of the modeling potential of iPSC (58, 59, 60). However, the combination of terminal endothelial cells and multipotential NSCs is still performing well and exhibiting unique advantages in NVU modeling, such as easier to use, lower cost, and especially promising in the exploration the interactions between neurogenesis (neural component) and angiogenesis (vascular component). Additionally, the cultures generated from adult stem cell tend to exhibit an adult phenotype. The protocol also tends to be shorter than those need to generate iPSC‐derived cultures, because the starting material is already a tissue‐specific progenitor. The investigation of NVU modeling with primary rodent cells allows direct testing of NVU and provides insights for exploring of the NVU in a human context. Such projects—combined with in vivo data on signaling molecules from rodents—will lead to the application of the NVU models to neurovascular‐based treatments for human patients (61).

Interestingly, after the transplantation of the 3D NVU to the infarcted side of the cerebral ischemia rats, it still had biological activity and could promote the neurogenesis and angiogenesis and reduce the infarcted volume of the ischemic side, which was not reported for other NVU models. These results indicate that the 3D NVU constructed from primary cells is functional and can be used to investigate neurogenesis and angiogenesis in cases of cerebral ischemia.

A recent study has employed in vitro BBB to model Alzheimer’s disease (57) and it displays one of promising practical application of in vitro BBB/NVU in brain‐related diseases modeling. Vessels and neurons both play vital roles in the development of the central nervous system and the occurrence, development, and prognosis of various diseases, such as stroke and Alzheimer’s disease. For ischemic stroke, neurovascular dysfunction is the main pathological character, e.g., a reduced capillary density, neuronal loss, and neural apoptosis, leading to insufficient oxygenation in infarct tissue and neurological deficits (62). In contrast to the traditional NVU model, our 3D NVU model not only has the structural characteristics of the vascular and neural components, but also is applicable to pathological studies: the pathological changes in the neuronal loss, vascular structures destruction, and BBB dysfunction under OGD are similar to those for the in vivo NVU under an ischemic stroke. Our data proved that those damages was mitigated by supplementing the VEGF. Additionally, the oxidative stress pathological symptoms, which is commonly induced by cerebral ischemic stroke, was also existed in OGD exposed 3D NVU but improved by edaravone. The proposed 3D NVU model can be employed to mimic the occurrence and development of cerebral ischemia and to investigate angiogenesis and neurogenesis under pathological or physiological conditions in vitro.

## 5 CONCLUSIONS

Primary NSCs and BMECs were co‐cultured in a 3D Matrigel matrix, which was a robust method for generating a 3D NVU with functional vascular‐like structures, BBB‐like characteristics, and diverse nerve cells, including neurons, astrocytes, and oligodendrocytes. This 3D NVU system facilitates the accurate physiological representation of the brain by mimicking the interplay between neural and vascular components as well as the complex in vivo cellular interactions and structures. Additionally, our 3D NVU model can be used to mimic the pathological changes and investigate angiogenesis and neurogenesis in the context of ischemia, using a well‐characterized in vitro OGD pattern. The proposed 3D NVU model also provides a valuable platform and precise spatiotemporal control for neurogenesis and angiogenesis research and the investigation of brain functions, which rely on interactions among brain cells, drug screening studies as a therapeutic strategy, and clinical applications (e.g., in the case of degenerative disease).

## AUTHOR CONTRIBUTIONS

Hongjin Wang designed and performed the experiments, analyzed the data, and wrote and revised the article. Huan Yang performed the in vitro experiments and analyzed the data. Yuhong Shi performed the experiments and analyzed the data. Yaping Xiao revised the manuscript and analyzed the data. Yue Yin, Baoxiang Jiang, and Huijing Ren analyzed the data. Weihai Chen and Qiang Xue provided financial support, reviewed, and revised the manuscript. Xiaoyu Xu designed the study, supervised the entire project, provided financial support, and reviewed the manuscript.

## CONFLICT OF INTEREST

All authors declare that there are no conflicts of interest.

## Supporting information

**FIGURE S1** Sketch of OGD pattern and VEGF, edaravone, or SU1498 treatment for 3D NVU

**FIGURE S2** Morphology and immunofluorescence characterization of primary NSCs and BMECs. (A) The primary NSCs proliferate and self‐renew to neurospheres and express specific markers NESTIN and SOX‐2, bar = 100 μm. (B) The primary BMECs exhibited long spindle‐shape morphology and presented a swirl monolayer, express specific markers CD31, OCCLUDIN, and ZO‐1, bar = 100 μm

**FIGURE S3** Dynamic gif figure of different fractions in 3D NVU. The different fractions of the 3D NVU were captured by using a confocal microscope (NIKON, A1+R10802)

**FIGURE S4** Dynamic gif figure of 3D NVU. The 3D reconstruction of the 3D NVU were captured by using a confocal microscope (NIKON, A1+R10802)

**FIGURE S5** GABAergic neurons and dopaminergic neurons in 3D NVU. Rabbit anti‐DAT (1:100, proteintech, 22524‐1‐AP) and rabbit anti‐GABRA1(1:100, proteintech,12410‐1‐AP) were used. Images were captured by using a confocal microscope (NIKON, A1+R10802). Bar = 100 μm

**FIGURE S6** Protective effects of edaravone on the OGD‐damaged 3D NVU. (A) Edaravone enhanced the cell viability of the OGD 3D NVU (data are presented as mean ± S.D., n = 3, ^*^ *p* < 0.05). (B) Edaravone reduced ROS in the 3D NVU (data are presented as mean ± S.D., n = 3, ^**^ *p* < 0.01). (C) Edaravone repressed the expression of superoxide anion in the 3D NVU (data are presented as mean ± SD, n = 3, ^**^ *p* < 0.01). (D) Edaravone increased the SOD concentration in the 3D NVU (data are presented as mean ± S.D., n = 3, ^*^ *p* < 0.05,^* *^ *p* < 0.01). (E) Edaravone reduced the MDA concentration in the 3D NVU (data are presented as mean ± S.D., n = 3, ^*^ *p* < 0.05). (F) Edaravone reduced the NO concentration in the 3D NVU. (G) Edaravone suppressed the leakage of LDH in the 3D NVU (data are presented as mean ± S.D., n = 3, ^**^ *p* < 0.01). (H‐J) Edaravone reduced the cell apoptosis rate in the 3D NVU (data are presented as mean ± S.D., n = 3, ^*^ *p* < 0.05, ^**^ *p* < 0.01)

Table S1‐S2
**Table S1** Antibodies used for immunofluorescence
**Table S2** Antibodies used for Western blotting

## Data Availability

The data used to support the findings of this study are available from the corresponding author upon request.

## References

[1] Potjewyd G, Moxon S, Wang T, Domingos M, Hooper NM. Tissue engineering 3D neurovascular units: a biomaterials and bioprinting perspective. Trends Biotechnol. 2018;36(4):457. 29422410 10.1016/j.tibtech.2018.01.003

[2] Da FA, Matias D, Garcia C, Amaral R, Geraldo LH, Freitas C, et al. The impact of microglial activation on blood‐brain barrier in brain diseases. Front Cell Neurosci. 2014;8:362. 25404894 10.3389/fncel.2014.00362 PMC4217497

[3] Mcconnell HL, Kersch CN, Woltjer RL, Neuwelt EA. The translational significance of the neurovascular unit. J Biol Chem. 2017;292(3):762–70. 27920202 10.1074/jbc.R116.760215 PMC5247651

[4] Nelson AR, Sweeney MD, Sagare AP, Zlokovic BV. Neurovascular dysfunction and neurodegeneration in dementia and Alzheimer’s disease. Biochim Biophys Acta. 2016;1862(5):887–900. 26705676 10.1016/j.bbadis.2015.12.016 PMC4821735

[5] Birgit O, Richard D, Ransohoff RM. Development, maintenance and disruption of the blood‐brain barrier. Nat Med. 2013;19(12):1584–96. 24309662 10.1038/nm.3407 PMC4080800

[6] Iadecola C. The neurovascular unit coming of age: a journey through neurovascular coupling in health and disease. Neuron. 2017;96(1):17. 28957666 10.1016/j.neuron.2017.07.030 PMC5657612

[7] Hongjin W, Han C, Baoxiang J, Shiqi Y, Xiaoyu X. Reconstituting neurovascular unit based on the close relations between neural stem cells and endothelial cells: an effective method to explore neurogenesis and angiogenesis. Rev Neurosci. 2020;31(2):143–59. 31539363 10.1515/revneuro-2019-0023

[8] Nakagawa S, Deli MA, Kawaguchi H, Shimizudani T, Niwa M. A new blood‐brain barrier model using primary rat brain endothelial cells, pericytes and astrocytes. Neurochem Int. 2009;54(3–4):253–63. 19111869 10.1016/j.neuint.2008.12.002

[9] Qiang X, Yang L, Hongyi Q, Qiang M, Ling X, Weihai C, et al. A novel brain neurovascular unit model with neurons, astrocytes and microvascular endothelial cells of rat. Int J Biol Sci. 2013;9(2):174–89. 23412420 10.7150/ijbs.5115 PMC3572400

[10] Bang S, Lee SR, Ko J, Son K, Tahk D, Ahn J, et al. A low permeability microfluidic blood‐brain barrier platform with direct contact between perfusable vascular network and astrocytes. Sci Rep. 2017;7(1):8083. 28808270 10.1038/s41598-017-07416-0 PMC5556097

[11] Brown JA, Pensabene V, Markov DA, Allwardt V, Neely MD, Shi M, et al. Recreating blood‐brain barrier physiology and structure on chip: a novel neurovascular microfluidic bioreactor. Biomicrofluidics. 2015;9(5):54124. 10.1063/1.4934713 PMC4627929 26576206

[12] Campisi M, Shin Y, Osaki T, Hajal C, Chiono V, Kamm RD. 3D self‐organized microvascular model of the human blood‐brain barrier with endothelial cells, pericytes and astrocytes. Biomaterials. 2018;180:117–29. 30032046 10.1016/j.biomaterials.2018.07.014 PMC6201194

[13] Osaki T, Sivathanu V, Kamm RD. Engineered 3D vascular and neuronal networks in a microfluidic platform. Sci Rep. 2018;8(1):5168. 29581463 10.1038/s41598-018-23512-1 PMC5979969

[14] Uwamori H, Higuchi T, Arai K, Sudo R. Integration of neurogenesis and angiogenesis models for constructing a neurovascular tissue. Sci Rep. 2017;7(1):17349. 29229920 10.1038/s41598-017-17411-0 PMC5725567

[15] Wang JD, Khafagy E, Khanafer K, Takayama S, Elsayed MEH. Organization of endothelial cells, pericytes, and astrocytes into a 3D microfluidic in vitro model of the blood‐brain barrier. Mol Pharm. 2016;13(3):895–906. 26751280 10.1021/acs.molpharmaceut.5b00805

[16] Boutin ME, Kramer LL, Livi LL, Brown T, Moore C, Hoffman‐Kim D. A three‐dimensional neural spheroid model for capillary‐like network formation. J Neurosci Methods. 2018;299:55–63. 28143748 10.1016/j.jneumeth.2017.01.014

[17] Cho C, Wolfe J, Fadzen CM, Calligaris D, Hornburg K, Chiocca EA, et al. Blood‐brain‐barrier spheroids as an in vitro screening platform for brain‐penetrating agents. Nat Commun. 2017;8(1):15623. 28585535 10.1038/ncomms15623 PMC5467173

[18] Chou CH, Sinden JD, Couraud PO, Modo M. In vitro modeling of the neurovascular environment by coculturing adult human brain endothelial cells with human neural stem cells. PLoS One. 2014;9:e106346. 25187991 10.1371/journal.pone.0106346 PMC4154686

[19] Urich E, Patsch C, Aigner S, Graf M, Iacone R, Freskgård PO. Multicellular self‐assembled spheroidal model of the blood brain barrier. Sci Rep. 2013;3:1500. 23511305 10.1038/srep01500 PMC3603320

[20] Zehendner CM, White R, Hedrich J, Luhmann HJ. A Neurovascular blood‐brain barrier in vitro model. Methods Mol Biol. 2014;1135:403–13. 24510882 10.1007/978-1-4939-0320-7_33

[21] Hatherell K, Couraud PO, Romero IA, Weksler B, Pilkington GJ. Development of a three‐dimensional, all‐human in vitro model of the blood–brain barrier using mono‐, co‐, and tri‐cultivation Transwell models. J Neurosci Methods. 2011;199(2):223–9. 21609734 10.1016/j.jneumeth.2011.05.012

[22] Abbott NJ, Rönnbäck L, Hansson E. Astrocyte–endothelial interactions at the blood–brain barrier. Nat Rev Neurosci. 2006;7(1):41–53. 16371949 10.1038/nrn1824

[23] Marinke VDH, Andries VDM, Eijkel J, Albert VDB, Segerink L. Microfluidic organ‐on‐chip technology for blood‐brain barrier research. Tissue Barriers. 2016;4(1):e1142493. 27141422 10.1080/21688370.2016.1142493 PMC4836466

[24] Sweeney MD, Ayyadurai S, Zlokovic BV. Pericytes of the neurovascular unit: key functions and signaling pathways. Nat Neurosci. 2016;19(6):771–83. 27227366 10.1038/nn.4288 PMC5745011

[25] Kisler K, Nelson AR, Montagne A, Zlokovic BV. Cerebral blood flow regulation and neurovascular dysfunction in Alzheimer disease. Nat Rev Neurosci. 2017;18(7):419–34. 28515434 10.1038/nrn.2017.48 PMC5759779

[26] Takano T, Tian GF, Peng W, Lou N, Nedergaard M. Astrocyte‐mediated control of cerebral blood flow. Nat Neurosci. 2006;9(2):260–7. 16388306 10.1038/nn1623

[27] Lancaster MA, Knoblich JA. Generation of cerebral organoids from human pluripotent stem cells. Nat Protoc. 2014;9(10):2329–40. 25188634 10.1038/nprot.2014.158 PMC4160653

[28] Yin X, Mead BE, Safaee H, Langer R, Karp JM, Levy O. Engineering stem cell organoids. Cell Stem Cell. 2016;18(1):25–38. 26748754 10.1016/j.stem.2015.12.005 PMC4728053

[29] Qin S, Goderie SK, Li J, Nithin K, Yu S, Natalia A, et al. Endothelial cells stimulate self‐renewal and expand neurogenesis of neural stem cells. Science. 2004;304(5675):1338–40. 15060285 10.1126/science.1095505

[30] Liu Y, Xue Q, Tang Q, Hou M, Qi H, Chen G, et al. A simple method for isolating and culturing the rat brain microvascular endothelial cells. Microvasc Res. 2013;90:199–205. 23978334 10.1016/j.mvr.2013.08.004

[31] Se Hoon C, Young Hye K, Matthias H, Christopher S, Seungkyu L, Carla D, et al. A three‐dimensional human neural cell culture model of Alzheimer’s disease. Nature. 2011;515(7526):274–8. 10.1038/nature13800 PMC4366007 25307057

[32] Cakir B, Xiang Y, Tanaka Y, Kural MH, Parent M, Kang Y, et al. Engineering of human brain organoids with a functional vascular‐like system. Nat Methods. 2019;16(11):1169–75. 31591580 10.1038/s41592-019-0586-5 PMC6918722

[33] Gemma L, Stefan R, Nikolaus P, Roland V, Arthur L. Modeling stroke in mice: permanent coagulation of the distal middle cerebral artery. J Vis Exp Jove. 2014;4(89):e51729. 10.3791/51729 PMC4692348 25145316

[34] Liu H, Hu Y, Zhang X, Na W, Peng X. Improved electrocoagulation method for establishing rat cerebral apoplexy model. J Third Mil Med Univ. 2011;33(17):1798–802.

[35] Chen Y, Constantini S, Trembovler V, Weinstock M, Shohami E. An experimental model of closed head injury in mice: pathophysiology, histopathology, and cognitive deficits. J Neurotrauma. 1996;13(10):557. 8915907 10.1089/neu.1996.13.557

[36] Shen LH, Li Y, Chen J, Zhang J, Vanguri P, Borneman J, et al. Intracarotid transplantation of bone marrow stromal cells increases axon‐myelin remodeling after stroke. Neuroscience. 2006;137(2):393–9. 16298076 10.1016/j.neuroscience.2005.08.092

[37] Anna H, van der Meer AD, FitzGerald EA, Park TE, Sleeboom JJF, Ingber DE. Distinct contributions of astrocytes and pericytes to neuroinflammation identified in a 3D human blood‐brain barrier on a chip. PLoS One. 2016;11(3):e0150360. 26930059 10.1371/journal.pone.0150360 PMC4773137

[38] Nico B, Frigeri A, Nicchia GP, Quondamatteo F, Herken R, Errede M, et al. Role of aquaporin‐4 water channel in the development and integrity of the blood‐brain barrier. J Cell Sci. 2001;114(7):1297–307. 11256996 10.1242/jcs.114.7.1297

[39] Tavazoie M, Van Der VL, Silva‐Vargas V, Louissaint M, Colonna L, Zaidi B, et al. A specialized vascular niche for adult neural stem cells. Cell Stem Cell. 2008;3(3):289–300. 18786415 10.1016/j.stem.2008.07.025 PMC6864413

[40] Richard D, Lu Z, Kebede AA, Barres BA. Pericytes are required for blood‐brain barrier integrity during embryogenesis. Nature. 2010;468(7323):562–6. 20944625 10.1038/nature09513 PMC3241506

[41] Paredes I, Himmels P, Carmen RDA. Neurovascular communication during cns development. Dev Cell. 2018;45(1):10–32. 29634931 10.1016/j.devcel.2018.01.023

[42] Ward NL, Lamanna JC. The neurovascular unit and its growth factors: coordinated response in the vascular and nervous systems. Neurol Res. 2004;26(8):870–83. 15727271 10.1179/016164104X3798

[43] Erik S, Diether L, Peter C. VEGF: once regarded as a specific angiogenic factor, now implicated in neuroprotection. BioEssays. 2004;26(9):943–54. 15351965 10.1002/bies.20092

[44] Ford MC, Bertram JP, Sara Royce H, Michael M, Qi L, Michael Y, et al. A macroporous hydrogel for the coculture of neural progenitor and endothelial cells to form functional vascular networks in vivo. Proc Natl Acad Sci U S A. 2006;103(8):2512–7. 16473951 10.1073/pnas.0506020102 PMC1413771

[45] Tarantini S, Tran CHT, Gordon GR, Ungvari Z, Csiszar A. Impaired neurovascular coupling in aging and Alzheimer’s disease: Contribution of astrocyte dysfunction and endothelial impairment to cognitive decline. Exp Gerontol. 2016;94:52–8. 27845201 10.1016/j.exger.2016.11.004 PMC5429210

[46] Abner L, Sudha R, Caroline L, Goldman SA. Coordinated interaction of neurogenesis and angiogenesis in the adult songbird brain. Neuron. 2002;34(6):945. 12086642 10.1016/s0896-6273(02)00722-5

[47] Li Y, Chang S, Li W, Tang G, Ma Y, Liu Y, et al. cxcl12 ‐engineered endothelial progenitor cells enhance neurogenesis and angiogenesis after ischemic brain injury in mice. Stem Cell Res Ther. 2018;9(1):139. 29751775 10.1186/s13287-018-0865-6 PMC5948880

[48] Li Q, Ford Eb M, Madri J. Modeling the neurovascular niche: VEGF‐ and BDNF‐mediated cross‐talk between neural stem cells and endothelial cells: an in vitro study. J Neurosci Res. 2010;84(8):1656–68. 10.1002/jnr.21087 17061253

[49] Yue H, Xie K, Ji X, Xu B, Wang C, Shi P. Vascularized neural constructs for ex‐vivo reconstitution of blood‐brain barrier function. Biomaterials. 2020;245:119980. 32229330 10.1016/j.biomaterials.2020.119980

[50] Rookmaaker MB, Schutgens F, Verhaar MC, Clevers H. Development and application of human adult stem or progenitor cell organoids. Nat Rev Nephrol. 2015;11:546–54. 26215513 10.1038/nrneph.2015.118

[51] Paschos NK, Brown WE, Eswaramoorthy R, Hu JC, Athanasiou KA. Advances in tissue engineering through stem cell‐based co‐culturele. J Tissue Eng Regen Med. 2015;9(5):488–503. 24493315 10.1002/term.1870

[52] Philp D, Chen SS, Fitzgerald W, Orenstein J, Margolis L, Kleinman HK. Complex extracellular matrices promote tissue‐specific stem cell differentiation. Stem Cells. 2010;23(2):288–96. 10.1634/stemcells.2002-0109 15671151

[53] Wagers AJ, Christensen JL, Weissman IL. Cell fate determination from stem cells. Gene Ther. 2002;9(10):606–12. 12032706 10.1038/sj.gt.3301717

[54] Kaisar M, Sajja R, Prasad S, Abhyankar V, Liles T, Cucullo L. New experimental models of the blood‐brain barrier for CNS drug discovery. Expert Opin Drug Discov. 2017;12(1):89–103. 27782770 10.1080/17460441.2017.1253676 PMC5521006

[55] Rahman NA, Rasil ANHM, Meydinglamade U, Craemer E, Diah S, Tuah AA, et al. Immortalized endothelial cell lines for in vitro blood–brain barrier models: a systematic review. Brain Res. 2016;1642:532–45. 27086967 10.1016/j.brainres.2016.04.024

[56] Srinivasan B, Kolli AR, Esch MB, Abaci HE, Shuler ML, Hickman JJ. TEER measurement techniques for in vitro barrier model systems. J Lab Autom. 2015;20(2):107. 25586998 10.1177/2211068214561025 PMC4652793

[57] Blanchard JW, Bula M, Davila‐Velderrain J, Akay LA, Tsai L‐H. Reconstruction of the human blood–brain barrier in vitro reveals a pathogenic mechanism of APOE4 in pericytes. Nat Med. 2020;26(6):952–63. 32514169 10.1038/s41591-020-0886-4 PMC7704032

[58] Gore A, Li Z, Fung HL, Young JE, Agarwal S, Antosiewiczbourget J, et al. Somatic coding mutations in human induced pluripotent stem cells. Nature. 2011;471(7336):63‐7. 21368825 10.1038/nature09805 PMC3074107

[59] Hussein SM, Batada NN, Vuoristo S, Ching RW, Autio R, et al. Copy number variation and selection during reprogramming to pluripotency. Nature. 2011;471(7336):58–62. 21368824 10.1038/nature09871

[60] Lister R, Pelizzola M, Kida YS, Hawkins RD, Nery JR, Hon G, et al. Hotspots of aberrant epigenomic reprogramming in human induced pluripotent stem cells. Nature. 2011;471(7336):68–73. 21289626 10.1038/nature09798 PMC3100360

[61] Otsuki L, Brand AH. The vasculature as a neural stem cell niche. Neurobiol Dis. 2017;107:4–14. 28132930 10.1016/j.nbd.2017.01.010

[62] Ye X, Asim M, Michael C. Angiogenesis, neurogenesis and brain recovery of function following injury. Curr Opin Investig Drugs. 2010;11(3):298. PMC2836170 20178043

