# Supplementary figures and images for: Reconstituting neurovascular unit with primary neural stem cells and brain microvascular endothelial cells in three‐dimensional matrix
Source: Brain Pathol. 2021 Feb 12;31(5):e12940. doi: 10.1111/bpa.12940 (PMC8412118; doi:10.1111/bpa.12940)

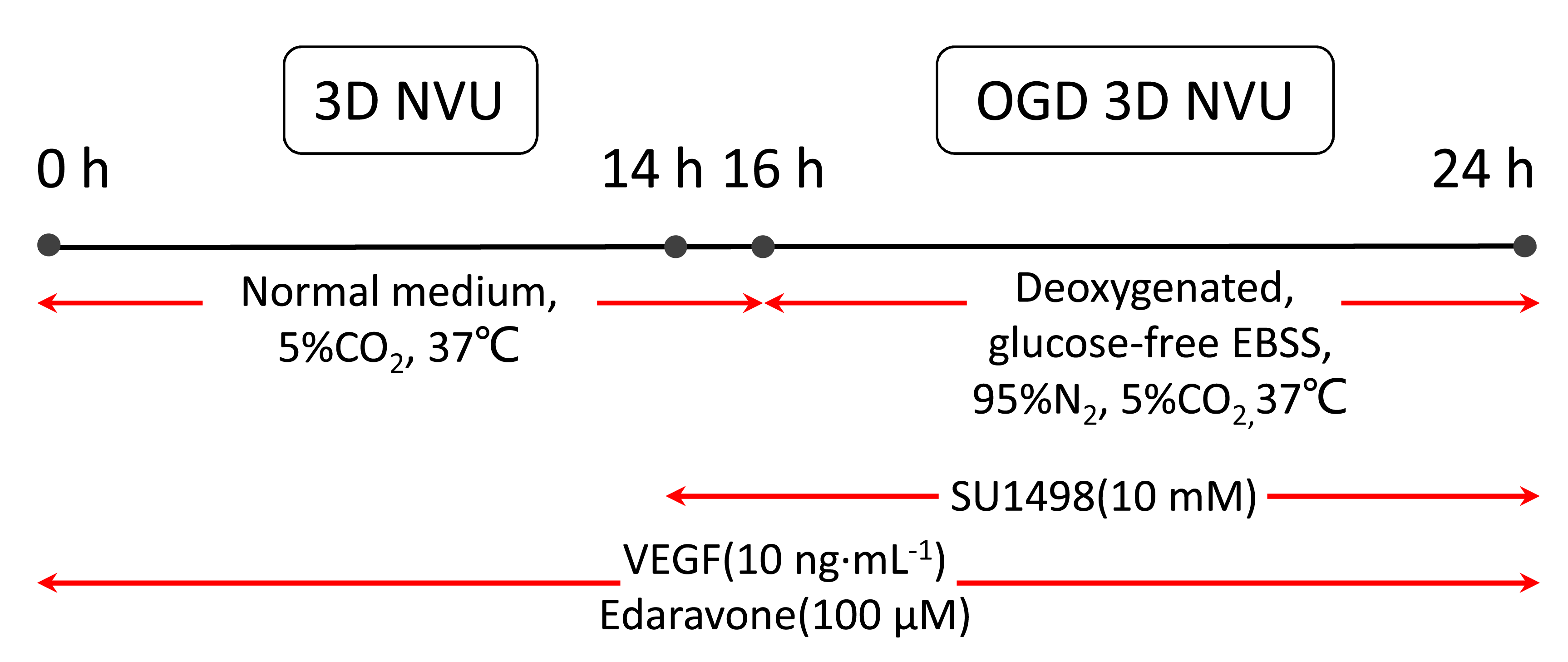

Supplement: Supplementary file 1 — FIGURE S1 Sketch of OGD pattern and VEGF, edaravone, or SU1498 treatment for 3D NVU [file BPA-31-e12940-s003.tif]

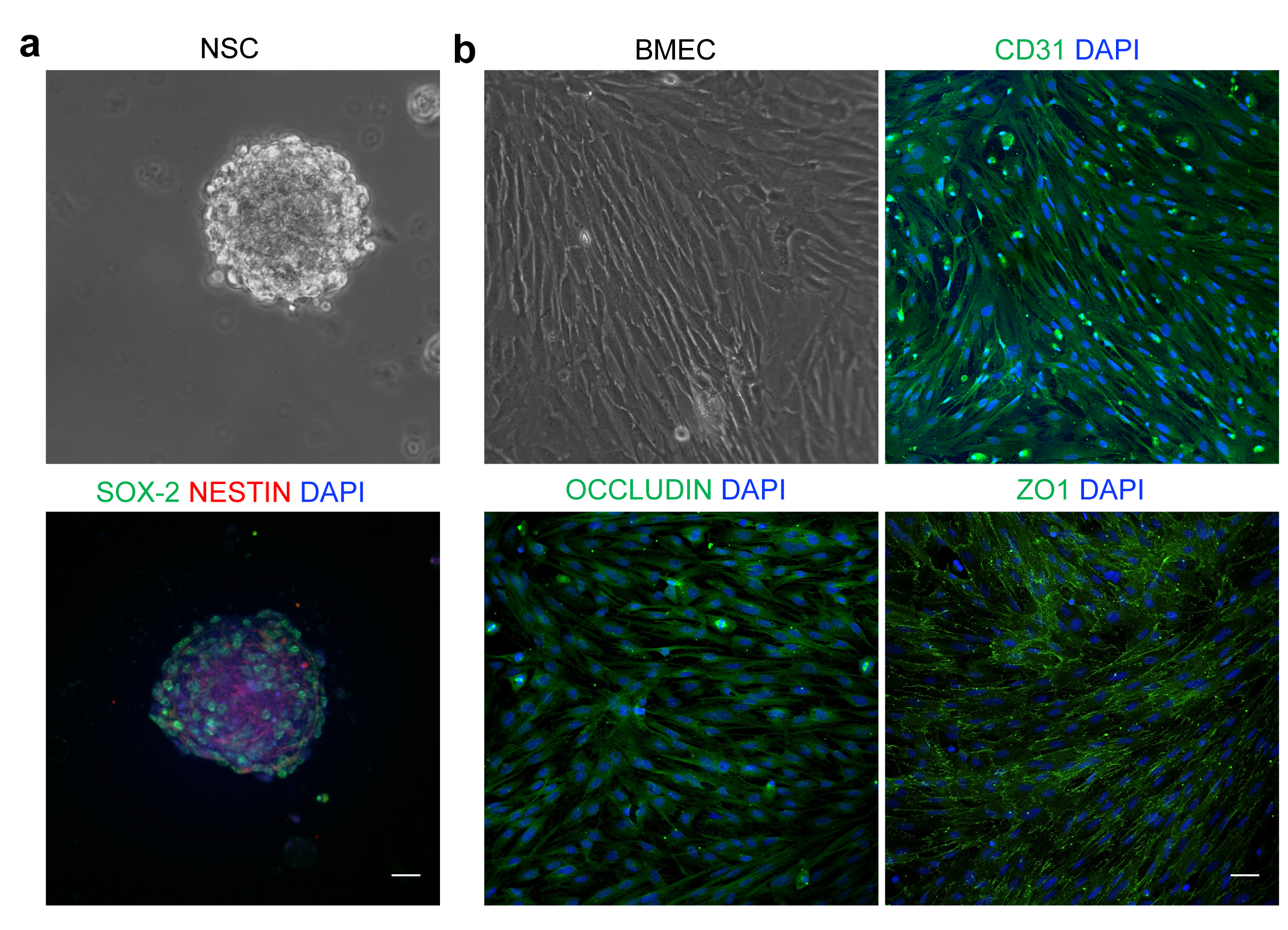

Supplement: Supplementary file 2 — FIGURE S2 Morphology and immunofluorescence characterization of primary NSCs and BMECs. (A) The primary NSCs proliferate and self‐renew to neurospheres and express specific markers NESTIN and SOX‐2, bar = 100 μm. (B) The primary BMECs exhibited long spindle‐shape morphology and presented a swirl monolayer, express specific markers CD31, OCCLUDIN, and ZO‐1, bar = 100 μm [file BPA-31-e12940-s002.tif]

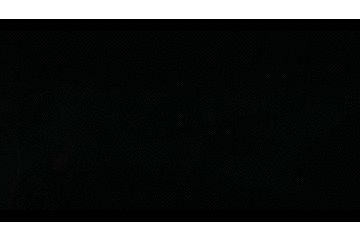

Supplement: Supplementary file 3 — FIGURE S3 Dynamic gif figure of different fractions in 3D NVU. The different fractions of the 3D NVU were captured by using a confocal microscope (NIKON, A1+R10802) [file BPA-31-e12940-s004.gif]

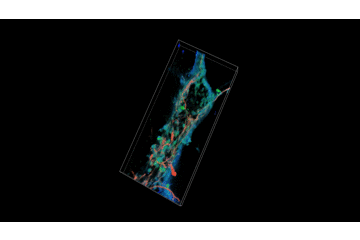

Supplement: Supplementary file 4 — FIGURE S4 Dynamic gif figure of 3D NVU. The 3D reconstruction of the 3D NVU were captured by using a confocal microscope (NIKON, A1+R10802) [file BPA-31-e12940-s001.gif]

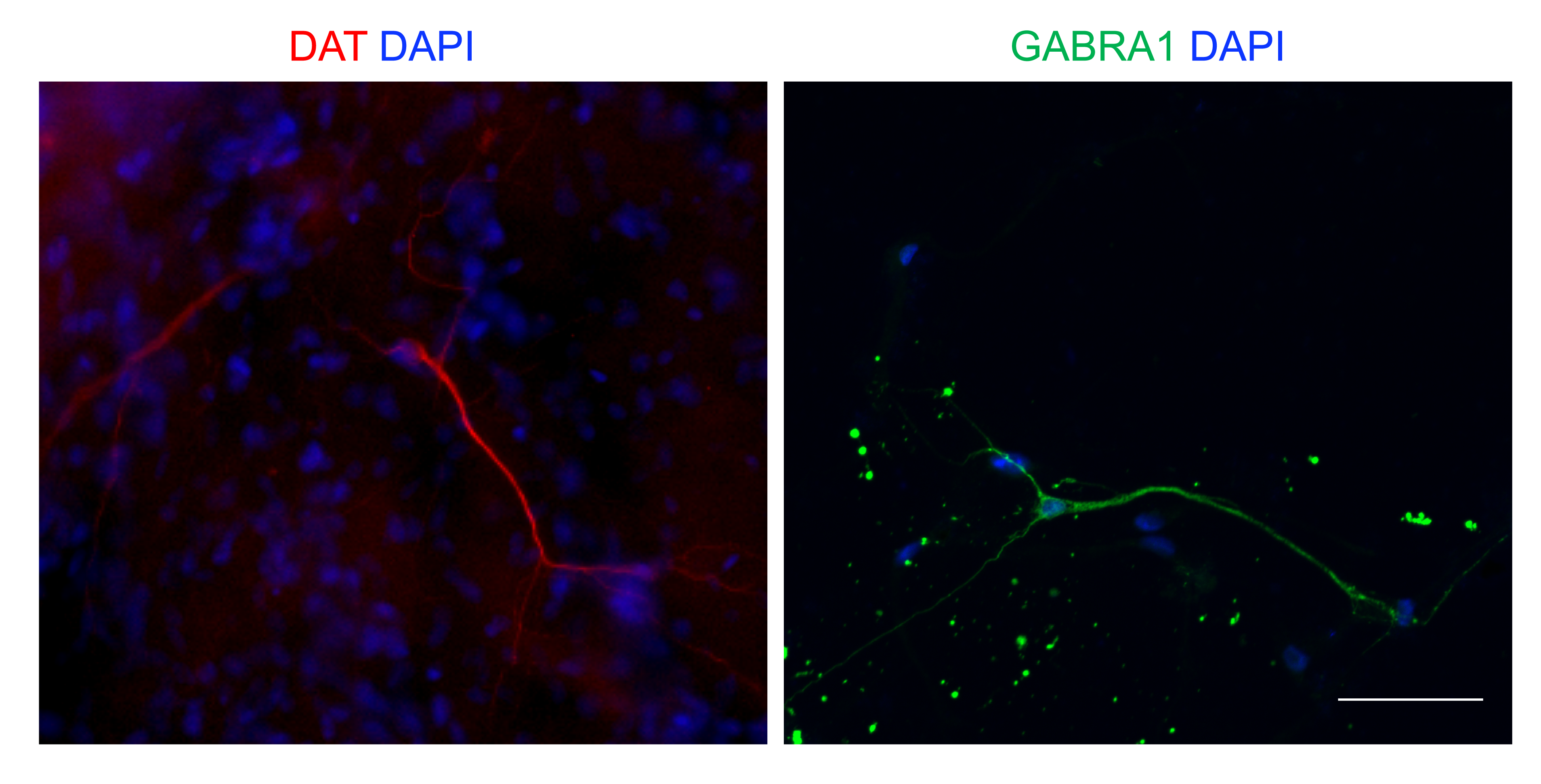

Supplement: Supplementary file 5 — FIGURE S5 GABAergic neurons and dopaminergic neurons in 3D NVU. Rabbit anti‐DAT (1:100, proteintech, 22524‐1‐AP) and rabbit anti‐GABRA1(1:100, proteintech,12410‐1‐AP) were used. Images were captured by using a confocal microscope (NIKON, A1+R10802). Bar = 100 μm [file BPA-31-e12940-s006.tif]

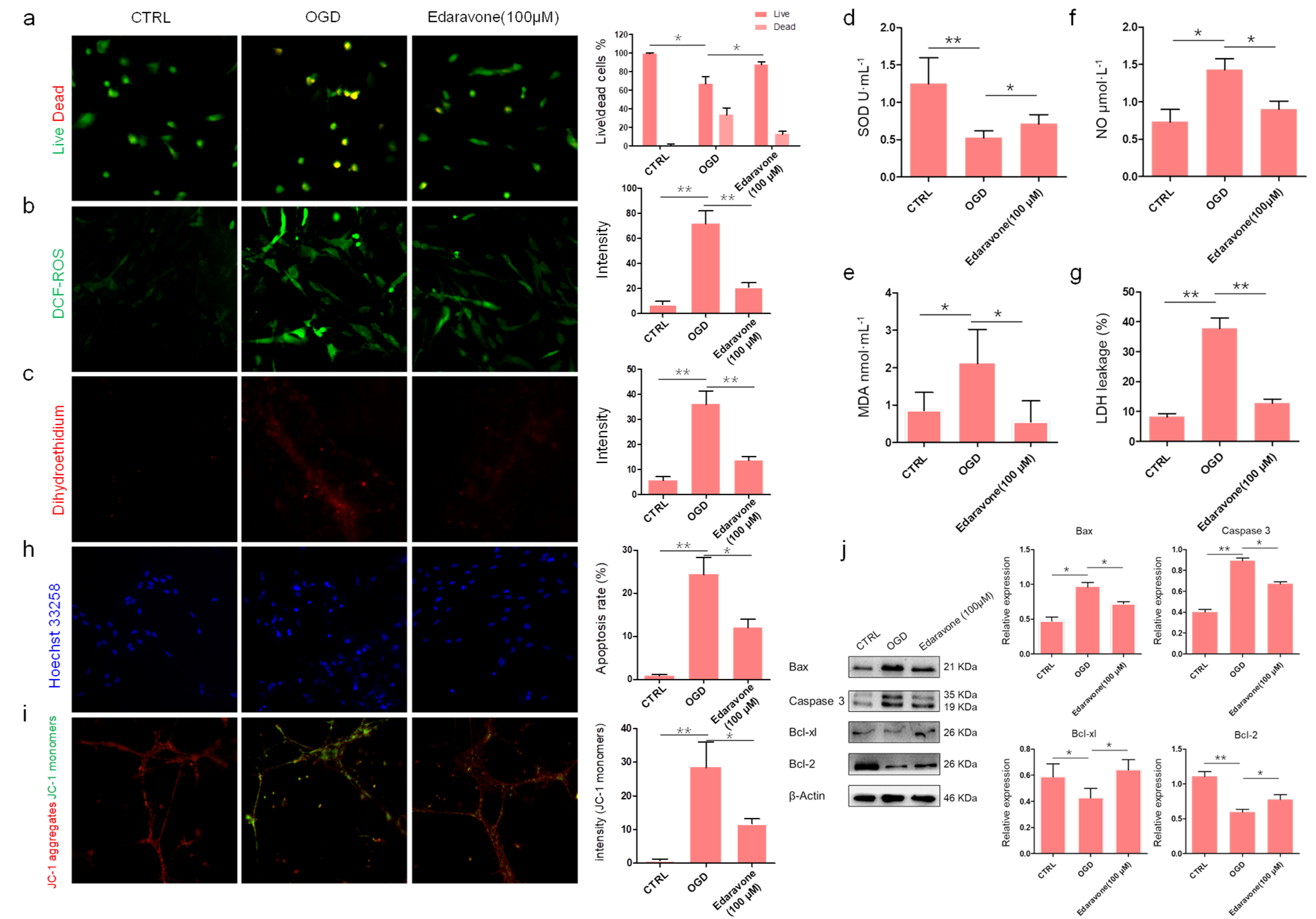

Supplement: Supplementary file 6 — FIGURE S6 Protective effects of edaravone on the OGD‐damaged 3D NVU. (A) Edaravone enhanced the cell viability of the OGD 3D NVU (data are presented as mean ± S.D., n = 3, * p < 0.05). (B) Edaravone reduced ROS in the 3D NVU (data are presented as mean ± S.D., n = 3, ** p < 0.01). (C) Edaravone repressed the expression of superoxide anion in the 3D NVU (data are presented as mean ± SD, n = 3, ** p < 0.01). (D) Edaravone increased the SOD concentration in the 3D NVU (data are presented as mean ± S.D., n = 3, * p < 0.05,* * p < 0.01). (E) Edaravone reduced the MDA concentration in the 3D NVU (data are presented as mean ± S.D., n = 3, * p < 0.05). (F) Edaravone reduced the NO concentration in the 3D NVU. (G) Edaravone suppressed the leakage of LDH in the 3D NVU (data are presented as mean ± S.D., n = 3, ** p < 0.01). (H‐J) Edaravone reduced the cell apoptosis rate in the 3D NVU (data are presented as mean ± S.D., n = 3, * p < 0.05, ** p < 0.01) [file BPA-31-e12940-s005.tif]
