# Supplementary material for: Reconstituting neurovascular unit with primary neural stem cells and brain microvascular endothelial cells in three‐dimensional matrix
Source: Brain Pathol. 2021 Feb 12;31(5):e12940. doi: 10.1111/bpa.12940 (PMC8412118; doi:10.1111/bpa.12940)
Supplement: Supplementary file 7 — Table S1‐S2 Table S1 Antibodies used for immunofluorescence Table S2 Antibodies used for Western blotting [file BPA-31-e12940-s007.docx]

**Supplementary tables and figures**

**STab. 1 Antibodies used for immunofluorescence**

| Useful markers for | Antigen | Host | Supplier | Cat.no. | Dilution |
| --- | --- | --- | --- | --- | --- |
| BMECs/cortical vessels | CD31 | Mouse | Santa Cruz | sc-46694 | 1:100 |
| BMECs | CD31 | Goat | R＆D | AF3628 | 1:20 |
| Astrocytes | GFAP | Rabbit | Proteintech | 60190-1 | 1:200 |
| Neurons | β3-tubulin | Mouse | CST | 4466 | 1:200 |
| Neurons | β3-tubulin | Rabbit | CST | 8572 | 1:200 |
| Oligodendrocytes | CNPase | Rabbit | CST | 8572 | 1:100 |
| Oligodendrocytes | MBP | Chicken | Abcam | ab123499 | 1:100 |
| NSCs | NESTIN | Mouse | CST | 4760 | 1:300 |
| NSCs | SOX-2 | Rabbit | Abcam | ab97959 | 1:100 |
| Tight junction | ZO1 | Rabbit | Proteintech | 21773-1 | 1:200 |
| Tight junction | OCCLUDIN | Rabbit | Proteintech | 13409-1-AP | 1:200 |
| Tight junction | Claudin-5 | Rabbit | Invitrogen | 341600 | 1:50 |
| Adherens Junction | VE-Cadherin | Rabbit | CST | 2158 | 1:100 |
| BBB-Like structures | P-gp | Rabbit | Bioss | bs-0563R | 1:100 |
| BBB-Like structures | GULT1 | Rabbit | Proteintech | 21829-1-AP | 1:100 |
| Endfeet | AQP4 | Rabbit | Bioss | bs-0634R | 1:200 |
| Vascular-like structures | LAMB1 | Rabbit | Proteintech | 23498-1-AP | 1:200 |
| Vascular-like structures | Vitronectin | Rabbit | Proteintech | 15833-1-AP | 1:100 |
| Vascular-like structures | ICAM-1 | Rabbit | Proteintech | 10020-1-AP | 1:100 |
| Proliferating cells | Ki67 | Rabbit | Biorbyt | orb389335 | 1:100 |
| Cortical neurons | NeuN | Rabbit | CST | 24307 | 1:100 |
| Alexa Fluor 405, 488,  594, and 647  secondary antibodies | Anti-rabbit, goat, mouse, and chicken | Donkey | Abcam/Jackson  ImmunoResearch | ab175651/ab150129/ ab150108/703-605-155 | 1:200 |
| Alexa Fluor 488, 594 secondary antibodies | Anti-rabbit, mouse | Goat | Abcam | ab150077/ab150116 | 1:200 |

**STab. 2 Antibodies used for Western blotting**

| Antigen | Host | Supplier | Cat.no. | Dilution |
| --- | --- | --- | --- | --- |
| GFAP | Rabbit | Proteintech | 60190-1 | 1:1000 |
| β3-tubulin | Mouse | CST | 4466 | 1:1000 |
| CNPase | Rabbit | CST | 8572 | 1:1000 |
| NESTIN | Mouse | CST | 4760 | 1:1000 |
| SOX2 | Rabbit | Abcam | ab97959 | 1:1000 |
| ZO1 | Rabbit | Proteintech | 21773-1 | 1:500 |
| OCCLUDIN | Rabbit | Proteintech | 13409-1-AP | 1:500 |
| Claudin-5 | Rabbit | Invitrogen | 341600 | 1:100 |
| AQP4 | Rabbit | Bioss | bs-0634R | 1:500 |
| LAMB1 | Rabbit | Proteintech | 23498-1-AP | 1:500 |
| Vitronectin | Rabbit | Proteintech | 15833-1-AP | 1:500 |
| ICAM-1 | Rabbit | Proteintech | 10020-1-AP | 1:500 |
| Ki67 | Rabbit | Biorbyt | orb389335 | 1:500 |
| Histione H3 | Rabbit | Bioss | bsm-33042M | 1:500 |
| Bcl-2 | Rabbit | Bioss | bs-0032R | 1:500 |
| Bcl-xl | Rabbit | Proteintech | 10783-1-AP | 1:500 |
| Bax | Rabbit | Proteintech | 50599-2-AP | 1:500 |
| Casepase-3 | Rabbit | Proteintech | 19677-1-AP | 1:500 |
| Peroxidase-conjugated Anti-rabbit, mouse secondary antibodies | Goat | Jackson ImmunoResearch  /Proteintech | 111-035-003/ SA00001-1 | 1:5000/  1:3000 |

**SFig.1 Sketch of OGD pattern and VEGF, edaravone, or SU1498 treatment for 3D NVU.**

**SFig.2 Morphology and immunofluorescence characterization of primary NSCs and BMECs.** a, The primary NSCs proliferate and self-renew to neurospheres and express specific markers NESTIN and SOX-2, bar=100 μm. b, The primary BMECs exhibited long spindle-shape morphology and presented a swirl monolayer, express specific markers CD31, OCCLUDIN, and ZO-1, bar=100 μm.

**SFig.3 Dynamic gif figure of different fractions in 3D NVU.** The different fractions of the 3D NVU were captured by using a confocal microscope (NIKON, A1+R10802).

**SFig.4 Dynamic gif figure of 3D NVU.** The 3D reconstruction of the 3D NVU were captured by using a confocal microscope (NIKON, A1+R10802).

**SFig.5 GABAergic neurons and dopaminergic neurons in 3D NVU.** Rabbit anti-DAT (1:100, proteintech, 22524-1-AP) and rabbit anti-GABRA1(1:100, proteintech,12410-1-AP) were used. Images were captured by using a confocal microscope (NIKON, A1+R10802). Bar=100 μm.

**SFig.6 Protective effects of edaravone on the OGD-damaged 3D NVU.** a. Edaravone enhanced the cell viability of the OGD 3D NVU (data are presented as *mean ±* S.D.*, n=3, ^*^p<0.05*). b, Edaravone reduced ROS in the 3D NVU (data are presented as mean ± S.D*., n=3, ^**^p<0.01*). c, Edaravone repressed the expression of superoxide anion in the 3D NVU (data are presented as *mean ± SD, n=3, ^**^p<0.01*). d, Edaravone increased the SOD concentration in the 3D NVU (data are presented as mean ± S.D*., n=3, ^*^p<0.05,^**^p<0.01*). e, Edaravone reduced the MDA concentration in the 3D NVU (data are presented as *mean ±* S.D.*, n=3, ^*^p<0.05*)**.** f, Edaravone reduced the NO concentration in the 3D NVU. g, Edaravone suppressed the leakage of LDH in the 3D NVU (data are presented as *mean ± S.D., n=3, ^**^p<0.01*). h, i, j, Edaravone reduced the cell apoptosis rate in the 3D NVU (data are presented as *mean ± S.D., n=3, ^*^p<0.05, ^**^p<0.01*).
